# Gut dsDNA virome shows diversity and richness alterations associated with childhood obesity and metabolic syndrome

**DOI:** 10.1016/j.isci.2021.102900

**Published:** 2021-07-24

**Authors:** Shirley Bikel, Gamaliel López-Leal, Fernanda Cornejo-Granados, Luigui Gallardo-Becerra, Rodrigo García-López, Filiberto Sánchez, Edgar Equihua-Medina, Juan Pablo Ochoa-Romo, Blanca Estela López-Contreras, Samuel Canizales-Quinteros, Abigail Hernández-Reyna, Alfredo Mendoza-Vargas, Adrian Ochoa-Leyva

**Affiliations:** 1 Departamento de Microbiologia Molecular, Instituto de Biotecnologia, Universidad Nacional Autonoma de Mexico, Avenida Universidad 2001, Cuernavaca, Morelos 62210, Mexico; 2 Unidad de Genomica de Poblaciones Aplicada a la Salud, Facultad de Quimica, UNAM/Instituto Nacional de Medicina Genomica (INMEGEN), Mexico City, Mexico; 3 Instituto Nacional de Medicina Genomica (INMEGEN), Mexico City, Mexico

**Keywords:** biological sciences, physiology, microbiology, virology, endocrinology, omics

## Abstract

Changes in the human gut microbiome are associated with obesity and metabolic syndrome, but the role of the gut virome in both diseases remains largely unknown. We characterized the gut dsDNA virome of 28 school-aged children with healthy normal-weight (NW, n = 10), obesity (O, n = 10), and obesity with metabolic syndrome (OMS, n = 8), using metagenomic sequencing of virus-like particles (VLPs) from fecal samples. The virome classification confirmed the bacteriophages' dominance, mainly composed of Caudovirales. Notably, phage richness and diversity of individuals with O and OMS tended to increase, while the VLP abundance remained the same among all groups. Of the 4,611 phage contigs composing the phageome, 48 contigs were highly prevalent in ≥80% of individuals, suggesting high inter-individual phage diversity. The abundance of several contigs correlated with gut bacterial taxa; and with anthropometric and biochemical parameters altered in O and OMS. To our knowledge, this gut phageome represents one of the largest datasets and suggests disease-specific phage alterations.

## Highlights

• The VLP abundance remained the same between normal weight and obesity groups
• Increased phage richness and diversity is linked to the disease
• Bacteriophages dominated the gut virome with high inter-individual diversity
• Differential phage abundances and prevalences are associated with the disease

## Graphical Abstract

**Figure:**
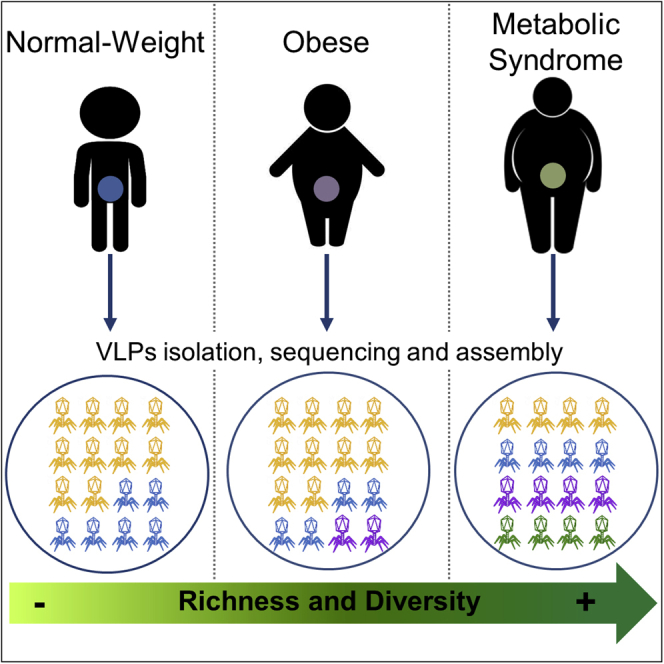

## Introduction

Childhood obesity (O) is one of the most relevant and severe health problems worldwide; it is a significant risk factor for severe infections, diabetes, and cardiovascular problems later in life. It is the leading cause of adult O, representing a substantial risk for premature death (Biro and Wien, 2010). It is considered a complex disease characterized by abnormal fat accumulation due to an imbalance between energy intake and expenditure (Romieu et al., 2017) that involves genetic, environmental, and lifestyle factors. In Mexico, 17.5% of school-aged children suffer from O (Romero-Martínez et al., 2019), placing it as the country with the world's second highest O rate (WHO, 2017). O is more than an accumulation of fat tissue, it involves chronic low-grade inflammation (Mattos et al., 2016), and it may be associated with metabolic disorders. These include high levels of blood glucose during fasting (hyperglycemia), elevated triglycerides (hypertriglyceridemia), low levels of the beneficial high-density lipoproteins (dyslipidemia), and high blood pressure (hypertension) (Evia-Viscarra et al., 2013). The metabolic syndrome diagnosis requires at least three of these conditions (De Ferranti et al., 2004), unfortunately highly prevalent in children with O (Perichart-Perera et al., 2007; Elizondo-Montemayor et al., 2013). Mexican children are considered a high-risk group (Romero-Martínez et al., 2019) for metabolic syndrome. Its prevalence has to lead to an increased incidence of type 2 diabetes mellitus (T2D) (DiBonaventura et al., 2018) and the development of cardiovascular disease (Franks et al., 2010) in adults.

The human gut microbiome is composed of a vast diversity of bacteria, archaea, and eukaryotic cells that together with viruses (mainly bacteriophages) comprise a diverse and complex ecosystem (Shkoporov and Hill, 2019). Some alterations in the gut microbiota are associated with an increased energy harvest from diet, low-grade inflammation, and altered adipose tissue composition (Pihl et al., 2016).These processes are considered the link between gut microbiota, O, and metabolic syndrome (Bouter et al., 2017). Several studies demonstrated microbiota alterations in O and obesity with metabolic syndrome (OMS) using 16S profiling (Bai et al., 2019; Chen et al., 2020; Gallardo-Becerra et al., 2020; Kim et al., 2020) and metatranscriptomic approaches (Gallardo-Becerra et al., 2020). It has been observed that dietary intervention was associated with changes in the virome community, in which individuals on the same diet converged (Minot et al., 2011). Changes in the virome structure due to a high-fat diet have been observed in mice, suggesting phage-host connections due to the microbial changes induced by diet (Kim and Bae, 2016).

Viral metagenomics is a relatively new and growing research field that studies the complete collection of viruses as part of the microbiota in any given niche (García-López et al., 2019).The gut virome is mainly dominated by bacteriophages (Shkoporov et al., 2019). It regulates the microbial ecosystem and host physiology (Virgin, 2014) through multiple interactions and the co-evolution with the host immune system (Barr et al., 2013), the bacteriome, and horizontal gene transfer events (Maiques et al., 2006). The viromic studies commonly use whole genome amplification (WGA) (Edwards and Rohwer, 2005; Brum and Sullivan, 2015) techniques to obtain a sufficient DNA amount to be sequenced. However, the multiple displacement amplification (MDA) has been associated with quantitative biases (Abulencia et al., 2006; Zhang et al., 2006; Yilmaz et al., 2010) and the preferential amplification of ssDNA viruses (Kim et al., 2008). The MDA-associated artifacts skew the community's taxonomic representation in non-repeatable ways and preclude quantitative analysis of viromes (Yilmaz et al., 2010). To overcome these biases, we used the tagmentation (TAG) method that supports a quantitative method for dsDNA with an ultra-low DNA input (Duhaime et al., 2012). Although the TAG method strongly selects against ssDNA templates (Edwards and Rohwer, 2005; Brum and Sullivan, 2015), 95% of the known human gut phageome is non-enveloped tailed dsDNA phages (Ofir and Sorek, 2018; Sausset et al., 2020).

Human disease-specific changes of the gut virome and phageome (the bacteriophage component of the virome) have been mainly reported in inflammatory bowel disease (Norman et al., 2015; Zuo et al., 2019), AIDS (Monaco et al., 2016), diabetes (Ma et al., 2018), and malnutrition (Reyes et al., 2015). However, studies addressing the role of the virome in O and metabolic syndrome have been limited to animal models (Kim and Bae, 2016; Rasmussen et al., 2020; Schulfer et al., 2020) and human adults (Minot et al., 2011; Manrique et al., 2021), overlooking children cohorts. Here, we characterized the dsDNA virome in ten healthy normal weight (NW) children, ten children with O, and eight children with OMS using metagenomic sequencing of virus-like particles (VLPs) from fecal samples. Our data show that alterations in the gut phageome are present in both O and OMS groups, providing the basis for diagnostic and therapeutic strategies based on phages for managing and preventing these conditions.

## Results

### The number of VLPs is similar between normal weight, obesity and obesity with metabolic syndrome groups

**Figure 1:**
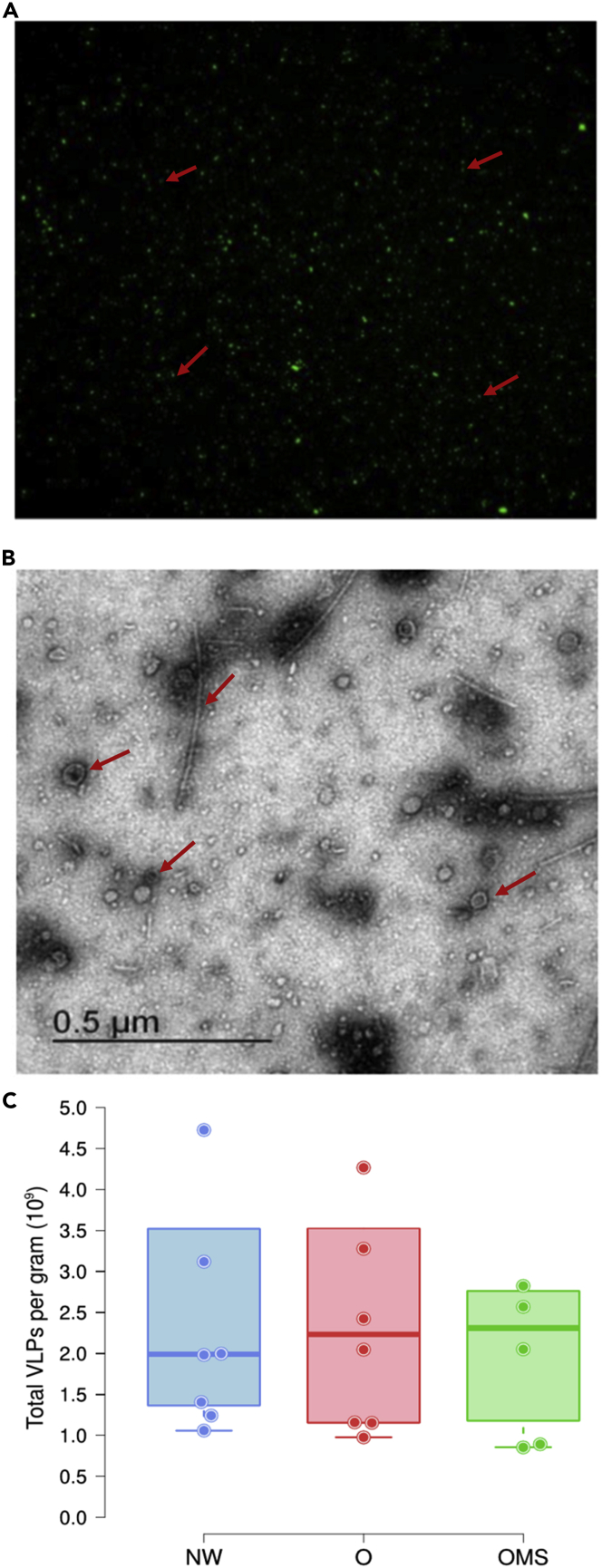
Microscopy visualization and counting of VLPs (A) SYBR Green I-stained virus-like particles (VLPs) assessed by epifluorescence microscopy. Red rows show an example of the VLPs. (B) TEM microscopy of VLPs. Red rows show an example of the VLP morphology. (C) The number of VLPs per gram of fecal matter for each group. Points represent the average number of VLPs for each sample. Error bars indicate the median and interquartile range, and differences were not significant (See Table S3).

We used 28 fecal samples previously collected from 7- to 10-year-old 10 healthy NW children, 10 children with O, and eight children with OMS, paired by gender and age (Table S1) and similar middle socioeconomic background (Gallardo-Becerra et al., 2020) (Table S2). The epifluorescence microscopy (Figure 1A) and the transmission electron microscopy (TEM) (Figure 1B) suggested the presence of VLPs in all samples. There was no significant difference in the number of VLPs among the three groups, obtaining an average of 2.56 x 10^9^, 2.85 x 10^9^, and 2.70 x 10^9^ VLPs for NW, O, and OMS groups per gram of feces, respectively (Figure 1C and Table S3).

### Functional profiles of the reads suggest viral presence in VLP samples

The DNA extracted from VLPs was treated with a TAG method for sequence library construction to avoid the bias of the WGA methods typically used due to the lower amount of DNA. After quality sequencing filters, we obtained an average of 4,871,075 paired-end sequences per sample, producing 11.23 Gb of data (Table S4). To only consider sequences potentially derived from VLPs, the reads mapped to bacterial (∼28%) and human (∼15%) genomes were discarded from further analyses (Table S4). The removal of bacterial sequences may lead to ignoring potential viral reads from prophages; nonetheless, we preferred to eliminate all potential bacterial DNA for this study. After that, we obtained 74,859,356 reads, an average of 2,673,548 reads per sample, with no significant differences in the sequence depth among the three groups (Figure S1 and Table S4). To obtain a first approach to the functional content of the VLP-derived reads, we annotated the reads using the KEGG database. As previously reported in virome studies (Breitbart et al., 2008; Reyes et al., 2010; Minot et al., 2011; Moreno-Gallego et al., 2019), most reads (96.2 ± 1.89%) mapped to genes with unknown functions (Figure S2). We also performed a search of the VLP-derived reads against the Prokaryotic Virus Orthologous Groups (pVOGs) and found that a small number of reads (1.93 ± 0.62%) matched with pVOGs (Figure S3), suggesting that sequences are of unknown viral origin.

### VLP-derived reads show an increased richness in the disease

We conducted 1,000 exercises of 149,000 randomly subsampled viral reads and clustered them at 95% identity to generate unique sequence clusters for each sample. In this way, we could analyze the sequence richness independent of the taxonomic classification and at the same sequence depth for all the samples. After that, we found an increase of unique sequences in OMS and O groups compared to the NW group (Figure S4), although this was not significant. This result suggests that the obesity groups (O and OMS) had increased viral reads richness compared to the NW.

### Bacteriophages dominated the viral reads of the gut virome

After clustering all the viral reads at 95% identity for each sample, there was a reduction of 68 ± 8% of sequences, resulting in an average of 856,825 unique sequences per sample (Table S4). As a first approach to obtain the potential viral content from the VLP-derived reads, we matched them against the viral non-redundant (NR) RefSeq and found that only 2.95 ± 0.95% had a match (Figure S5A). From these, 66.95 ± 6.95% of the reads matched to prokaryotic viruses, 6.79 ± 3.91% to eukaryotic viruses, and 26.26 ± 5.79% to an undefined classification including unknown viruses or multiple hits between eukaryotic and prokaryotic viruses (Figure S6).

### Virome assembly confirms the dominance of bacteriophages

Considering that the length of a phage genomes could affect the abundance obtained from the sequencing reads (Moreno-Gallego et al., 2019), we performed a *de novo* assembly using the 74,859,356 viral reads from all samples. To avoid chimeric contigs, we collected those contigs covering ≥80% of their total size by the viral reads in at least one sample, resulting in 18,602 contigs (≥500 nt; largest = 176,210 nt; N50 = 7,480 nt, Table S5). On average, 58.69 ± 12.79% of the viral reads from each sample mapped back to these contigs, showing a homogeneous contribution of all samples to the assembly (Figure S7 and Table S6). Next, we removed all short contigs (<4 kb) to remove potential fragmented viral genomes. After that, 12,287 contigs (N50 = 9,097 nt, Table S5) were obtained and used for further analyses. Importantly, this reduction in the number of contigs did not lead to a drastic decrease in the read recruitment, remaining on average 54.85 ± 14.60% of the viral reads from each sample (Figure S7 and Table S6). We found, on average, 7.20 genes per contig (0.87 genes per kb of contig length). We classified these contigs using the DNA and their encoding proteins, obtaining 4,611 contigs as potential prokaryotic viruses, 1,540 as potential eukaryotic viruses, 2,696 contigs with multiple hits to prokaryotic and eukaryotic viruses, and 3,440 contigs with an unknown origin. From the 4,611 prokaryotic contigs (≥ 4Kb) representing complete or partial genomes, 1,307 were classified using the DNA sequences (dc_megablast) and 3,304 using their encoded proteins (BLASTx). On the other hand, we classified the 12,287 contigs using VirSorter, obtaining a viral classification for 1,542 of them. Contrary, 5,949 contigs had a match with a pVOG. Interestingly, most classified contigs using VirSorter (69.58%) and pVOG (77.16%) coincide with our prokaryotic virus classification (Figure S8), reinforcing our phage classification strategy using both DNA and proteins. Previous virome studies reported that between 29.35% and 48.5% of the viral contigs mapped to a pVOG (Coutinho et al., 2019; Deboutte et al., 2020).

On average, the contig classification revealed 56.30 ± 5.50% of prokaryotic viruses and 13.94 ± 2.80% of eukaryotic viruses per sample. These were similar to the taxonomy classification obtained from reads (Figure S6), suggesting that the sequence diversity observed with reads was also captured in the assembled contigs. As expected by using a TAG method, most of the prokaryotic contigs were dsDNA viruses (96%), whereas only 0.1% of them were annotated as ssDNA viruses, and the remaining 4% were unclassified bacterial viruses. We did not observe any influence in the number of reads versus the number of viral contigs obtained per sample (Figure S9).

### The children's gut phageome was mainly composed of Caudovirales

The 4,611 contigs (N50 = 10,370 nt, max 176,210 nt; mean 9,347 nt, Table S5) classified as potential prokaryotic viruses were selected as the phageome and represented 37.53% of the total contigs of the virome assembly, hereafter mentioned as phage contigs. The phageome recruited more than half of the viral reads (50.27%) mapped to the original virome assembly and were distributed in an average of 707,571 reads per sample (Table S6).

Caudovirales composed the majority of phage contigs (91.28 ± 0.10%), followed by crAss-like viruses (0.64 ± 0.73%) (Figure S10). The high number of contigs classified as Caudovirales coincides with the high number of viral reads also classified as Caudovirales (Figure S5B), suggesting that our genome assembly also reflects the taxonomy of reads by themselves. Within Caudovirales contigs, the most abundant families were Siphoviridae (35.28 ± 0.02%), Myoviridae (31.33 ± 0.03%), and Podoviridae (6.50 ± 0.01%).

Given the importance of crAss-like phages in adult viromes (Dutilh et al., 2014; Shkoporov et al., 2018b), we analyzed their presence in our phageome, and we found that an average of 0.64 ± 0.01% of the contigs could be classified as crAss-like phages (Table S7). On the other hand, we found that the Mexican crAssphage (Cervantes-Echeverría et al., 2018) was present in 25 out of 28 samples.

### Increased richness, diversity, and dominance of phage contigs is linked to the shift from normal weight to obese

**Figure 2:**
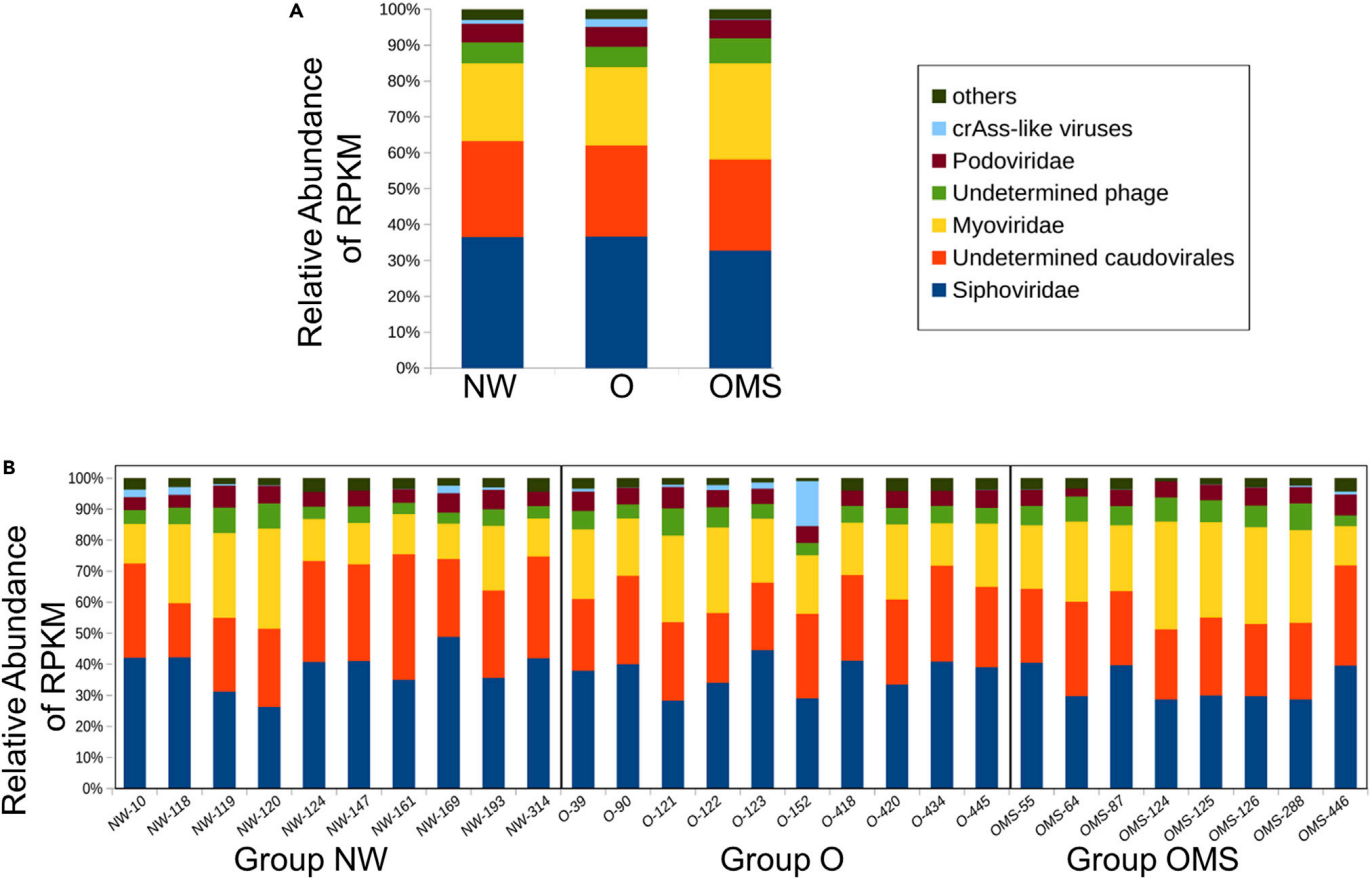
Relative abundance of normalized RPKM for the 4,611 phage contigs (A and B) (A) Average per group and (B) average per sample. “Others” represents the relative abundance of the less abundant phage contigs.

We normalized abundance by reads per kilobase per million (RPKM)-sequenced reads per sample to compare groups' phage abundance and diversity metrics (Reyes et al., 2015).After that, we compared the taxonomy abundances of classified phages among our groups (Figure 2). We found a decreased abundance of Siphoviridae and crAss-like phages (32.72 and 0.18%, respectively) in the OMS group as compared to O (36.66 and 2.2%, respectively) and NW (36.50 and 1.0%, respectively) groups. We also observed an increased abundance for Myoviridae in the OMS group (26.72%) as compared to O (21.77%) and NW (21.70%) groups. However, these abundances were not significantly different among the three groups.

**Figure 3:**
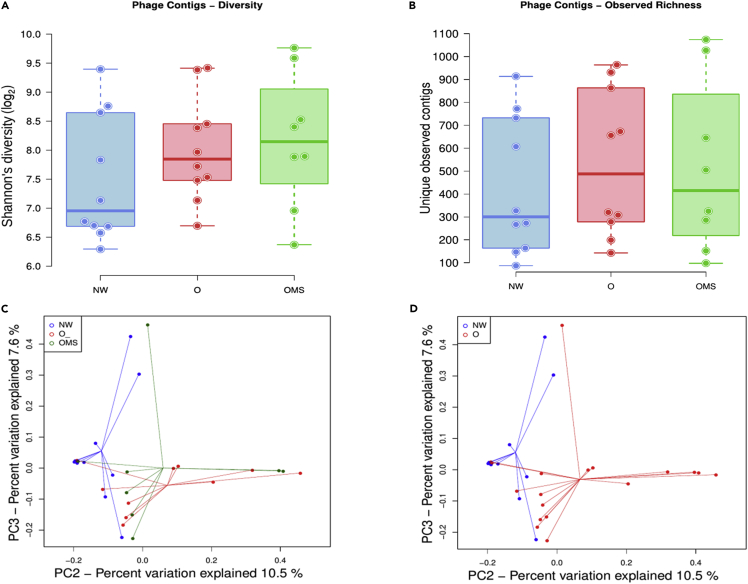
Alpha and beta diversity of the phageome (A) Phage contigs diversity. Each point shows the median of 10,000 Shannon diversity calculations at an even depth for a single sample based on even resampling, with boxes showing the group's distribution group. (B) Phage contigs richness. Each point shows the median of 10,000 observed contigs calculations at an even depth for a single sample based on even resampling, with boxes showing the group's distribution. Differences in (A) and (B) were not significant. Error bars represent the mean ± SD. (C) Principal coordinates analysis (PCoA) based on Bray-Curtis dissimilarity with samples tagged as NW, O, and OMS. (D) PCoA based on Bray-Curtis dissimilarity with samples tagged by all obese (O + OMS) in red circles and NW (See Figures S11 and S12).

The α-diversities of the phageome showed that phage richness and Shannon diversity increased in O and OMS groups compared to the NW group (Figures 3A and 3B), although the differences among groups were not significant. Notably, these changes in diversity happened maintaining a similar number of VLP counts in the three groups (Figure 1C). The O group exhibited the highest richness, followed by OMS and NW groups (Figure 3B), whereas the OMS group exhibited the highest diversity, followed by O and NW groups (Figure 3A). Notably, this increased richness in obese groups was also supported by our initial viral reads clustering analysis (Figure S4). We also observed that 488 (10.58%) phage contigs accounted for 70% of the normalized reads in the NW samples, whereas 679 (14.73%) and 831 (18.02%) phage contigs accounted the 70% of the normalized reads in O and OMS groups. These results suggest a considerable increase in the number of dominant phage contigs due to the disease.

On the other hand, the principal coordinates analysis based on Bray-Curtis distances showed no self-consistent clusters by the study group (Figures 3C and S11). However, when all the obese samples were tagged together (O + OMS), they cluster separately from NW samples (Figures 3D and S11). Although, this difference was not significant (p = 0.568). We also performed the Aitchison distance analysis subjected to dimensional reduction with a principal components analysis and showed non-significant separated clusters (Figure S12).

### Several phage contigs were significantly over-abundant in obese and metabolic syndrome groups

**Figure 4:**
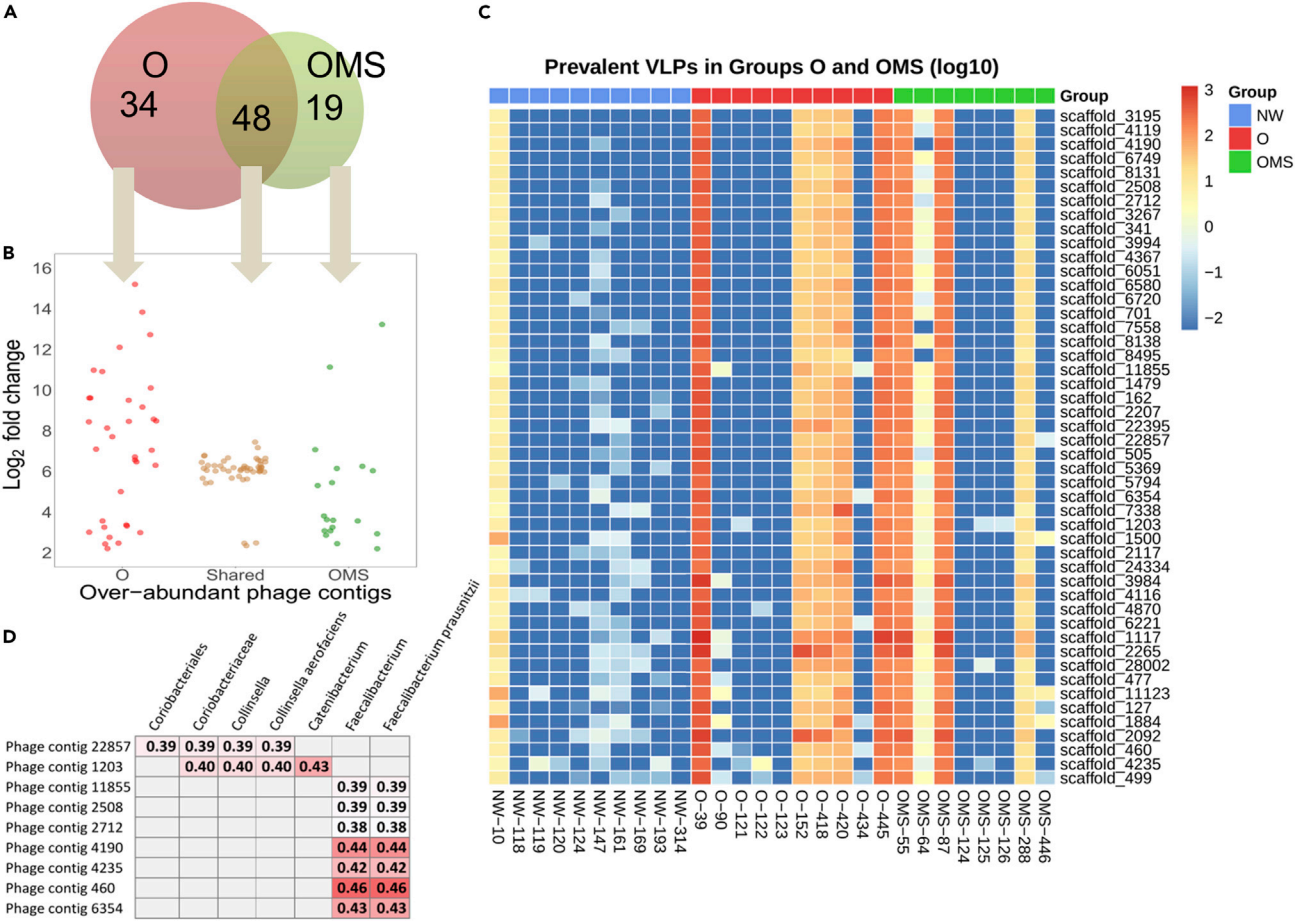
Analysis of over-abundant phage contigs in the obesity (O) and obesity with metabolic syndrome (OMS) (A) Venn diagram of over-abundant phage contigs in the O (red circle) and OMS (green circle) groups, as compared with the NW group. (B) Expression levels (>2 of log_2_ fold change) of over-abundant phage contigs from the Venn diagram. The 34 (red points), 19 (green points), and 48 (brown points) phage contigs of O, OMS, and shared among two groups, respectively. (C) Heatmap of the normalized RPKM abundances of the 48 phage contigs over-abundant in O and OMS groups. We show the abundance distribution for each contig among all samples. (D) Spearman correlation plots of the phage abundances (RPKM) of the 48 phage contigs over-abundant in the disease and the relative abundance of 16S bacterial taxa identified to be significantly associated with obesity. Only significant correlations with unadjusted p values (≤0.05) were displayed. See also Figure S13.

We identified the significantly over-abundant phage contigs in O and OMS groups compared to the NW group using all the phages in the normalized abundance matrix. This procedure is analogous to using tables of RPKM for the measurement of differentially expressed genes in RNA-seq experiments. After that, we obtained 111 and 107 phage contigs significantly over-abundant in O and OMS groups, respectively, and the differences were statistically significant at an alpha = 0.05 compared to the NW group. From those, we only selected the phage contigs shared in at least 30% of the O or OMS groups to eliminate the individual phages. This resulted in 82 and 67 phage contigs significantly over-abundant in O and OMS groups, respectively, compared to the NW group, with 48 phage contigs shared between O and OMS groups (Figures 4A and 4B).

We next assessed whether the abundance of the 48 phage contigs (Figure 4C) was associated with a parallel change in bacterial populations on all the samples. To answer this question, we selected the 16s rRNA gene sequencing data of 41 bacterial taxa significantly associated with O and metabolic syndrome (Gallardo-Becerra et al., 2020) and calculated the Spearman correlation between them and the abundance of the 48 phage contigs in all the samples. We found that the abundance of 9 phage contigs correlated with bacterial abundances associated with O and metabolic syndrome (Figure 4D). The samples that led to the correlation between phage contigs and bacteria were the O and OMS groups (Figure S13). We conducted the same correlation analysis for the 34 and 19 over-abundant phages of OMS and O groups (Figure 4A). However, the correlations were mainly caused by outlier samples and zero abundance values for most phage contigs (data not shown). The 19 phage contigs that were only over-abundant in the OMS group may be further studied as biomarkers linked to the development of metabolic syndrome in children with O.

### The phageome was mainly individual specific

**Figure 5:**
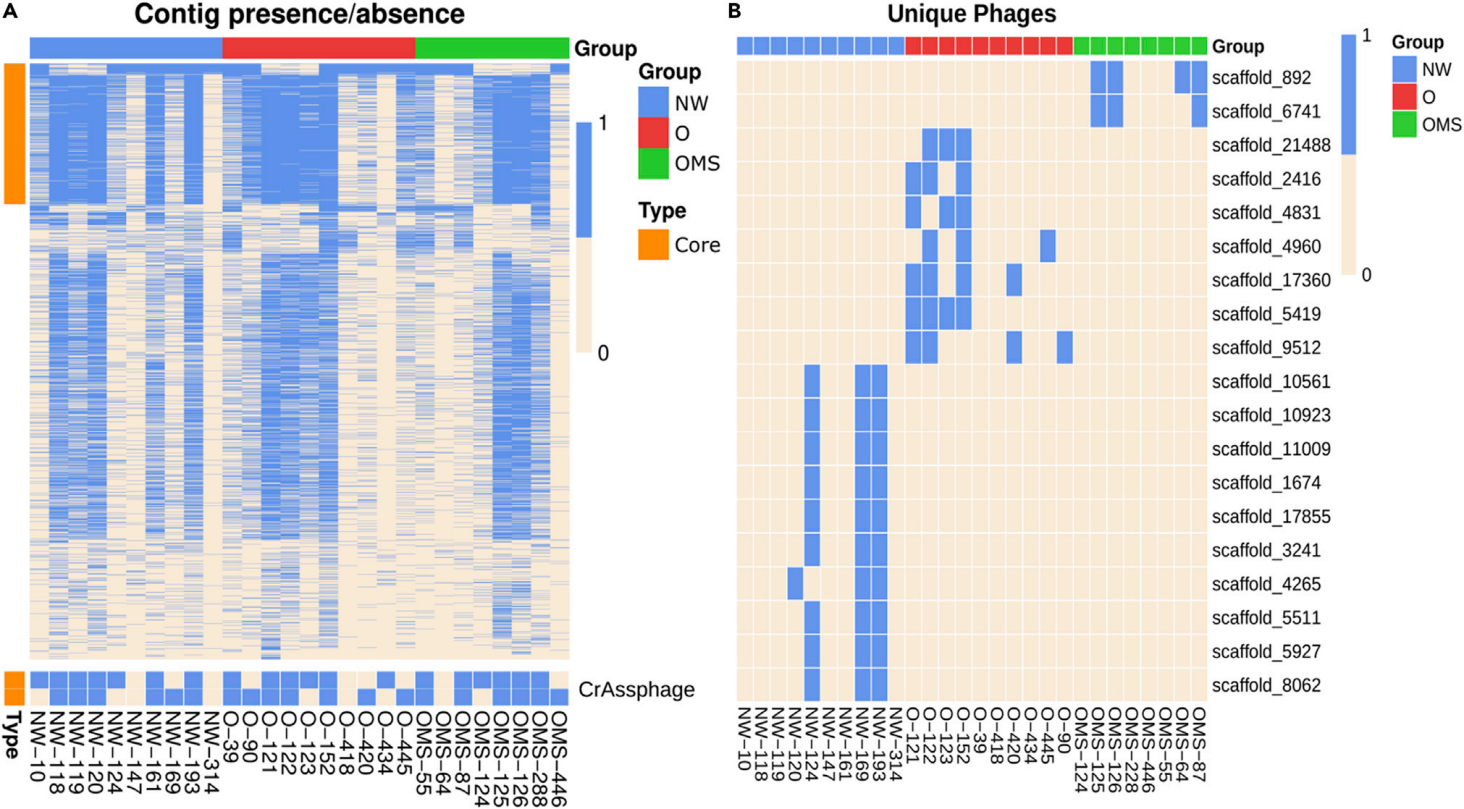
Presence-absence heat maps of the phage contig distributions among all samples (A) Distribution of core phage contigs among all the samples. crAssphage-like contigs from the core group are presented separately at the bottom. (B) Unique phages for each group.

The recruitment matrix was analyzed to assess the phageome composition in all the samples independently of the health status. From the 4,611 phage contigs, only two were present in all 28 individuals and 48 in more than 23 individuals (>80%), which were named the core phages. The majority of phage contigs (3,477) were shared in less than 14 individuals (50% of the population) (Figure 5A). These results suggest that most of the phageomes were individual specific, being only 48 phages (1.04% of phageome) considered core phages. Additionally, two of the “core phages” were identified as putative “crAss-like” phages. The presence of “crAss-like" phages as part of the core supports our definition for core phages as these are the most abundant phages reported in the adult human gut (Dutilh et al., 2014).

We did not find unique group-specific phage contigs in 100% of the samples and that they were absent in the other groups. It coincides with the high inter-individual phage presence. To find possible unique phages, we looked at the phage contigs using different cut-offs of phage presence among the population, from ≥20 to ≥80% of the samples in one group and were absent in all samples of the other groups. We found that only using a cut-off of contigs with ≥30% obtained the maximum numbers of unique contigs, obtaining two unique phage contigs in the OMS group, seven unique phage contigs in the O group, and ten unique phage contigs in the NW group (Figure 5B).

### The disease altered the prevalence of highly abundant NW phage contigs

We compared the prevalence of the phage contigs with a higher presence (>80% of samples) in NW to O and OMS samples. We found that 52 phage contigs were present in >80% of NW samples with an average prevalence of 91.54%. In contrast, their prevalence was significantly reduced to 76.35% and 68.27% in O and OMS groups, respectively (p value= <0.0001) (Figure S14). These results showed that the prevalence of phage contigs with the highest presence in the NW group was significantly altered in O and OMS groups.

### The abundance of “core phages” correlated with bacterial taxa associated with obesity and metabolic syndrome

We assessed whether the 48 core phage contigs were associated with parallel changes in bacterial populations. To this end, we calculated the Spearman correlation between the 16s rRNA gene sequencing data of 41 bacterial taxa and the abundance of 48 core phage contigs in all the samples. After that, we only selected the Spearman correlations with r^2^ > 0.3 and unadjusted p value ≤0.05.

**Figure 6:**
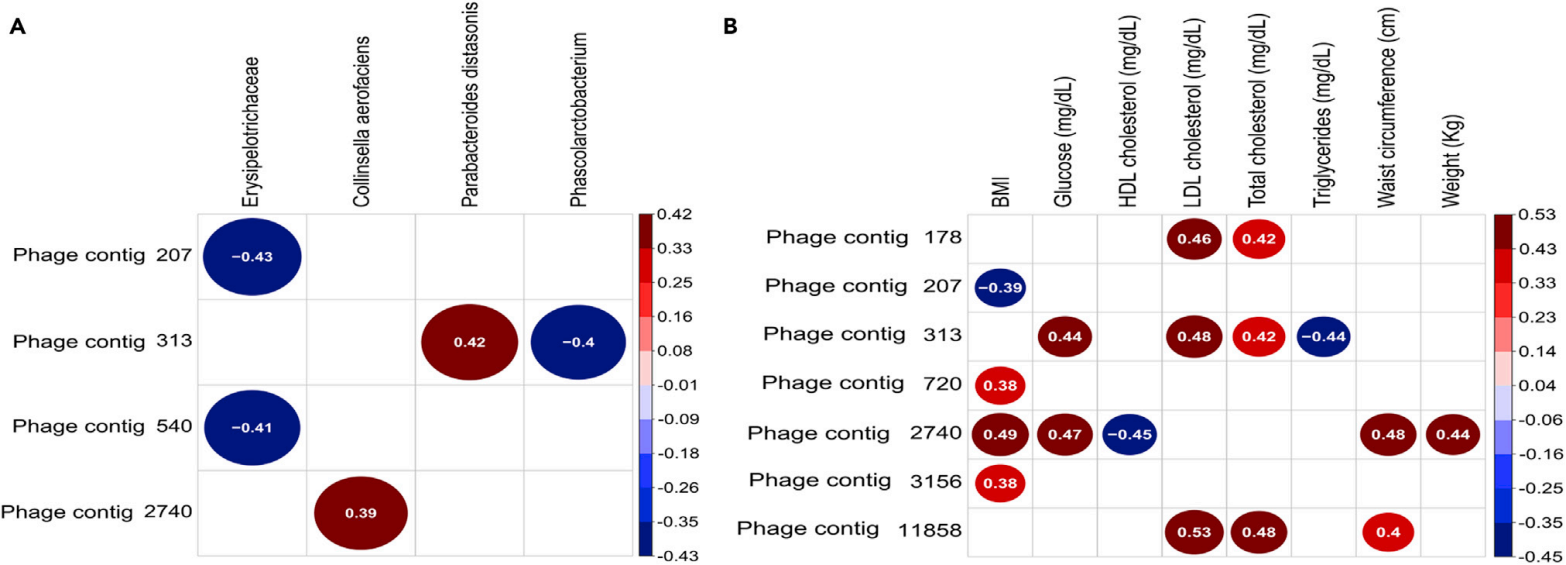
Disease-specific bacteria-phageome patterns in obesity and metabolic syndrome (A) Spearman correlation plots of the phage abundances (RPKM) of the 48 phage contigs with a higher prevalence (≥80% of all the samples) and the relative abundance of 16S bacterial taxa identified to be significantly associated with the disease. (B) Spearman correlation plots of the phage abundances (RPKM) of the 48 phage contigs with a higher prevalence (≥80% of all the samples) and the clinical and anthropometrical parameters altered in obesity and metabolic syndrome. Only significant correlations with unadjusted p values ≤0.05 were displayed. See also Figures S15 and S18.

The abundance of four phage contigs was correlated with bacterial abundances (Figures 6A and S15). Phage contig 2740, which was more prevalent in O than NW group (Figure S16A), was positively correlated with the abundance of *Collinsella aerofaciens* (Figures 6A and S15), a prevalent bacteria in the OMS group. Phage contig 313, more abundant in O vs. OMS group (Figure S16A), showed a positive correlation with *Parabacteroides distasonis*, also prevalent in O compared to OMS group, and a negative correlation with an undetermined species from genus *Phascolarctobacterium* (more abundant in NW vs. O) (Figures 6A and S15). Furthermore, the phage contig 313 showed high similarity (99.4% nt identity) with previously reported *Bacteroides* plasmids (sequence ID: CP059857.1 and AP019726.1). Phage contigs 207 and 540 had a lower prevalence in OMS and O groups than the NW group (Figure 6A), and both contigs were negatively correlated with Erysipelotrichaceae, an over-abundant family in O and OMS groups (Figures 6A and S15). These data might imply that some of the changes in the abundances of specific phage-bacteria interactions may be partly associated with the bacterial changes found in O and metabolic syndrome.

We also assessed whether these core phage contigs were associated with a parallel change in all bacterial populations, independently of the bacterial association to O and metabolic syndrome. To this end, we selected all the taxa of the 16s rRNA gene sequencing and calculated the Spearman correlation between them and the abundance of these core phage contigs in all the samples. Only the abundance of 30 phage contigs correlated with bacterial abundances associated with O and metabolic syndrome (Figure S17).

### The abundances of core phage contigs correlated with anthropometric and biochemical parameters altered by obesity and metabolic syndrome

We also analyzed whether the 48 core phage contigs correlated with the anthropometric and clinical parameters typically altered in obesity and metabolic syndrome, such as high body mass index (BMI), low levels of high-density lipoprotein (HDL), high levels of triglycerides, high glucose level, high waist circumference, and high weight (Gallardo-Becerra et al., 2020). The Spearman correlation between the 48 phage contigs and the anthropometrical and biochemical parameters, including all the samples of our study, showed positive correlations with several phage contigs (Figures 6B, S18, and S16B), suggesting an association between these phage contigs and anthropometric and biochemical parameters. We also found a negative correlation between the abundance of several phage contigs (Figure S18) and BMI, HDL levels, and triglycerides (Figures 6B and S18).

## Discussion

Metagenomic analysis of viruses, one of the most poorly understood components of the human gut microbiome, has recently revolutionized our view of the gut microbiome highlighting the critical role of the interactions between phages and bacteria in health and disease (Breitbart et al., 2003; Bikel et al., 2015; Garmaeva et al., 2019).

Here, we report a large-scale sequencing viral metagenome where instead of solely using the sequencing reads, we assembled the phageome community, obtaining 4,611 phage contigs ≥ 4 Kb representing complete or partial genomes. We used this size threshold to avoid having partial viral genomes and decrease the selection of eukaryotic viruses, mainly composed of the Anelloviridae family, with a reported genome size between 3 and 4 Kb (Reyes et al., 2015) and considering that shortest phage genomes are ≥ 4 Kb (Hatfull, 2008). Most virome studies use MDA phi29 WGA (Sutton and Hill, 2019) which unevenly amplifies linear genome fragments and preferentially amplifies small circular ssDNA viruses, e.g., the Microviridae family (Roux et al., 2016). This methodology probably limits statistical analyses of the viral community composition to presence-absence observation because relative abundances might be biased toward specific viruses. In contrast, we used a TAG method free of WGA. Although this method may now select against ssDNA (Yilmaz et al., 2010; Roux et al., 2016), it allowed us to perform analyses associated with the abundance of dsDNA phages, the major components of the human gut virome (Kim et al., 2011). Probably because of the TAG method, the abundance of Microviridae in our samples was much smaller than that previously found in most virome studies (Norman et al., 2015; Shkoporov et al., 2018b), highlighting the need for quantitative analysis protocols in virome studies, as was recently described (Roux et al., 2016).

The TAG method impacted the proportion of both Microviridae (0.031%) and of Inoviridae (0.137%) to a lesser extent (Table S7) since both families are ssDNA viruses. We do not know why the method affected the abundance of Microviridae more than Inoviridae. However, given that we used the same protocols for all samples, we expected the same bias for both families. The selection against ssDNA templates had been observed before in mock community experiments (Kim and Bae, 2011; Roux et al., 2016), favoring ssDNA viruses to be systematically under-represented (>10-fold) in TAG and over-represented (<10-fold) in WGA-obtained viromes (Roux et al., 2016). To avoid this bias, we decided to eliminate all ssDNA viruses from our analysis.

The Caudovirales were the most abundant phage order in our three groups. This predominance is consistent with that observed in previous human gut viromes (Norman et al., 2015; Reyes et al., 2015; Manrique et al., 2016). We showed that children with O and OMS had specific changes in their gut phageomes, particularly in the abundance of specific phage contigs. Importantly, the recruited children had similar lifestyles, the same ethnographic region, and relatively homogeneous environments, making the effects of socioeconomic, cultural, and nationality not confounding factors. We consider this feature to minimize bias in our results, as reported in previous studies (He et al., 2018; Stagaman et al., 2018; Zhong et al., 2019). Notably, we found a similar number of VLPs among the groups, independently of the disease status. Contrary, previous reports on Crohn disease have observed that sick patients harbored significantly more VLPs than healthy individuals (Norman et al., 2015).

We observed an increased diversity and richness in OMS and O groups than NW group, although it was not significant, probably to the limited sample size. This suggests that the expansion of specific phages in O and metabolic syndrome could decrease the presence of others, maintaining similar VLP amounts independently of the disease status. Interestingly, both sequencing reads and metagenomic viral assembly supported the increased richness observed in O and OMS groups. We previously observed a significant increase in bacterial richness and diversity in O and metabolic syndrome groups than the healthy NW group using the same set of samples used for the virome (Gallardo-Becerra et al., 2020).The increase of richness and diversity of phages and the bacteria associated with O and metabolic syndrome agrees with the recent proposal that the virome diversity is associated with adults' intestinal bacterial diversity (Moreno-Gallego et al., 2019).

An increase of viral richness and diversity has also been reported in Crohn disease, ulcerative colitis, and murine colitis (Duerkop et al., 2018). Contrary, low viral richness and diversity were detected in individuals with type 1 diabetes mellitus (Zhao et al., 2017). The phage contigs found at high prevalence in NW individuals were significantly lower in individuals with O and OMS. This would agree with a recent study in ulcerative colitis and Crohn disease that also found a reduction of phage contigs typically abundant in healthy individuals (Manrique et al., 2016). These results suggest that the loss of some phages with high prevalence in the NW group could be associated with O and metabolic syndrome.

We found a higher diversity and richness associated with OMS than in obese individuals, indicating the importance of studying the Phageome in these pathologies as separated diseases. Furthermore, we also found specific over-abundant phages for O group different from OMS group. There is a wide-ranging heterogeneity among individuals with O regarding their risk for developing metabolic dysfunction and its attendant complications (Viveiros and Oudit, 2020). The gut microbiota is involved in the etiology of O and O-related complications such as nonalcoholic fatty liver disease, insulin resistance, and T2D (Pedersen et al., 2016; Canfora et al., 2019), suggesting the importance of studying O separately from metabolic syndrome.

In this regard, recent studies suggest that the microbiota from metabolically healthy individuals with O could transition to OMS (Yuan et al., 2021), and a metatranscriptome and 16S profiling demonstrated significant differences between O and metabolic syndrome (Gallardo-Becerra et al., 2020). The abundance of some metabolism-related bacteria was associated with circulating inflammatory compounds in individuals with O without metabolic syndrome, suggesting that gut microbiota changes in metabolically healthy children with O conceivably serve as a compensatory response to a surfeit of nutrients (Yuan et al., 2021). The 19 phage contigs that were only over-abundant in the OMS group may be further studied as biomarkers linked to the development of metabolic syndrome in children with O. It would be interesting to validate this assumption by analyzing phage contigs at the functional level (Turnbaugh et al., 2009) and analyzing the over-abundant unique phages' functional profile in O and OMS groups.

We detected a high inter-individual variability of the phageome, with 75.41% of phage contigs detected in less than 50% of the individuals. In contrast, only 48 (1.04%) phage contigs were present in >80% of the individuals (core phages) independently of the disease. This observation agrees with previous evidence of a reduced healthy core in the human gut phageome, composed only of 23 phages (Manrique et al., 2016). The concept of a reduced core virome is still controversial. It has received support from a recent study with adult monozygotic twins, in which only 18 contigs were found in all individuals (Moreno-Gallego et al., 2019). In contrast, the compilation of a large-scale gut virome database called into question the existence of a human core gut virome (Gregory et al., 2019; Shkoporov et al., 2019). Our findings on a reduced number of shared phages support the idea of a highly individual-specific gut virome (Shkoporov et al., 2018b; Moreno-Gallego et al., 2019; Shkoporov et al., 2019) and a high inter-individual viral diversity (Reyes et al., 2010; Minot et al., 2013; Shkoporov et al., 2019) because only 1% of our viral assembly (4611 phage contigs) belongs to core phage contigs. This percentage is similar to previously reported core contigs, where 0.6% of the viral assembly (3639 viral clusters) (Shkoporov et al., 2019) and 1.3% of the viral assembly (1703 viral contigs) (Manrique et al., 2016) were shared among the majority of individuals.

According to previous reports, contamination with some bacteria from different extraction kits (including ours) was reported (Laurence et al., 2014; Salter et al., 2014), mostly are soil- or water-dwelling bacteria frequently related with nitrogen fixation, probably associated with nitrogen that is often used instead of air in ultrapure water storage tanks (Kulakov et al., 2002). However, we did not find reports about viral contamination. Unlike high-biomass samples (such as from feces), kit contamination is a particular challenge for low-biomass studies (such as from blood or the lung), which may provide little template DNA to compete with that in the reagents for amplification (Tanner et al., 1998; Willerslev et al., 2004), suggesting that if there was contamination in our samples, it would be minimal and this should not be of significance on our results.

The criteria used to define the presence of a viral sequence in a sample (Gregory et al., 2019) are still questionable. According to the previous reports (He et al., 2018; Zhong et al., 2019) and our results, we suggest that the highly homogeneous geographic and ethnic representation across our dataset of samples was an essential factor that allowed us to establish a human core phageome, being this highly reduced with only 48 phage contigs. We followed similar sequence thresholds recently proposed for accurate estimation of viral community composition and diversity (Reyes et al., 2015), such as (i) contig length ≥4 kb, (ii) coverage determined from reads mapped at ≥90% identity, and (iii) ≥80% of contig length with ≥1× coverage. However, we should note that our assemblies may represent fragments of the same phage genome or family. More extensive studies using stringent standard bioinformatics parameters, such as previously suggested (Roux et al., 2017), are necessary to generate more accurate estimates about the core phageome and its prevalence in the human population. We suggest that the use of only contigs covering ≥80% of their total size by the viral reads in at least one sample decreased the rate of reads aligned (∼58%) to the initial virome assembly. Removing all shorter contigs (<4 kb) did not lead to a drastic reduction in reads recruitment, remaining ∼54% of the viral reads aligned. Similar reads recruitment percentage (49.66%) was obtained in a recent human gut virome using an extensive post-assembly decontamination process (Shkoporov et al., 2018b). Compelling evidence supports the importance of studying the virome concurrently with the bacteriome to obtain a holistic picture of the gut ecosystem changes in a disease such as inflammatory bowel disease (IBD) (Norman et al., 2015; Cornuault et al., 2018), where phage abundance was associated with changes in the abundance of specific gut bacterial species (Reyes et al., 2013). Also, fecal metabolomics in mice revealed that phage predation in the mouse gut microbiota could potentially impact the mammalian host by changing the levels of key metabolites involved in essential functions for the host (Hsu et al., 2019). According to these, we assessed whether the highly abundant phage contigs were associated with a parallel change in bacterial populations and the clinical parameters significantly altered in O and metabolic syndrome. Although the biochemical and anthropometric parameters reflected the difference between groups according to the disease, this group separation also correlated with several abundances of phages. In this regard, we observed that the abundance of phage contig 2740 positively correlated with *Collinsella aerofaciens*, low-density lipoprotein (LDL), BMI, waist circumference, glucose, weight, and lower HDL levels.

Further, *Collinsella aerofaciens* was significantly over-abundant in the OMS group (Gallardo-Becerra et al., 2020) and showed a positive correlation with triglycerides and a negative correlation with HDL in the same sample set. However, *Collinsella* is a highly abundant taxon in 12-month-old breastfed infants. It was previously reported as a signature of the developing anaerobic infant microbiome and could be involved in acquiring crAss-like phages (Bäckhed et al., 2015; Siranosian et al., 2020). These results suggest that the gut virome is also altered along with the microbiome in children with O. The phage contig 313 positively correlated with *Parabacteroides distasonis*, high LDL, glucose, and total cholesterol levels. This bacteria was enriched in O vs. OMS groups, and it was associated with increased LDL levels in the cohort of our study (Gallardo-Becerra et al., 2020) and weight gain and hyperglycemia in other studies (Wang et al., 2019).

The phage contig 313 showed high similarity with previously reported Bacteroides plasmids (sequence ID: CP059857.1 and AP019726.1). Because Bacteroides and Parabacteroides are closely related bacteria, these results suggest these groups of bacteria as putative hosts. Moreover, phage contig 313 was positively correlated with *Parabacteroides distasonis*. Together, these results suggest that this phage could be opting for a pseudo-lysogenic cycle and multiply with their hosts (Shkoporov et al., 2018a) since the replication strategy of crAss-like bacteriophages is that it co-replicates with its host in a way that does not disrupt the host proliferation (Shkoporov et al., 2018a). On the other hand, the high similarity of contig 313 with previously reported *Bacteroides* plasmids follows this group of bacteria as putative hosts. Unfortunately, little is known about the diversity of plasmids and pseudo-lysogenic bacteriophages in these groups of bacteria. However, more studies are needed to understand better viral replication that would help interpret these relationships. In contrast, the phage contig 313 showed a negative correlation with the genus Phascolarctobacterium, enriched in the NW vs. O groups (Gallardo-Becerra et al., 2020). This suggests that the increased abundance of this phage contig in O could be inhibiting the abundance of Phascolarctobacterium, potential protective bacteria against O (Gallardo-Becerra et al., 2020). Also, the increased abundance of phage contigs 207 and 540 in the NW group correlated with a decreased abundance of Erysipelotrichaceae, a bacterial family significantly increased in the OMS group and positively correlated with waist circumference (Gallardo-Becerra et al., 2020). A high abundance of Erysipelotrichaceae has been associated with host dyslipidemia in O, metabolic syndrome, and hypercholesterolemia (Spencer et al., 2011). It suggests that phage contigs 207 and 540 could be used to diminish the abundance of Erysipelotrichaceae in O and OMS groups (Gallardo-Becerra et al., 2020).

These examples open the possibility of using phages as a therapeutic option against the bacterial changes typically associated with O. Indeed, it has been suggested that gut phageome represents a source of individual phages with potential therapeutic applications (Ott et al., 2017). Even successful treatments against *Clostridium difficile* using bacteria-free fecal filtrate provided the first evidence that phageome manipulation may be an effective therapeutic strategy to stabilize the bacterial eubiosis in the microbiome (Ott et al., 2017). Compelling evidence supports the idea that shifts in the microbial system during infancy may increase the risk of O later in life (Scheepers et al., 2015). Therefore, manipulating the gut microbiota using phages at an early stage might offer the prevention and treatment of the bacterial changes associated with O. Fecal microbiota transplantation revealed that phages could be co-transferred with bacteria (Draper et al., 2018).

We believe that our study provides a better knowledge of the phage-bacteria interactions in the gut microbiome. The development of *in vivo* models to test the phage-bacteria dynamics in O will undoubtedly be an essential area that could help complement the understanding of phage's role in microbiota changes associated with O and metabolic syndrome.

### Limitations of the study

This study could be limited by the number of studied samples, which probably accounted for the lack of statistical significance obtained in some of the analyses. We performed an accurate estimation of viral community composition and diversity. However, we should note that our assemblies may represent fragments of the same phage genome that could affect accurate estimates about our phageome and its prevalence in the human population.

## STAR★Methods

### Key resource table

**Table:** 

REAGENT or RESOURCE	SOURCE	IDENTIFIER
**Chemicals, peptides, and recombinant proteins**		

RNA later	Thermo Fisher Scientific	Cat. AM7020
SM Buffer	Nalgene	Cat. 7252520
DNase I	Thermo Fisher Scientific	Cat. 18047019
SYBR Green	Thermo Fisher Scientific	Cat. K0221

**Critical commercial assays**		

QIAamp MinElute Virus Spin Kit	Qiagen	Cat. 57704
Qubit	Thermo Fisher Scientific	Cat. Q32851
Illumina Nextera XT DNA Library	Illumina	Cat. FC-131-1024

**Deposited data**		

Assembled contigs	This paper	BioProject PRJNA646512 BioSample SAMN15545081

**Software and algorithms**		

Trim Galore 1.12	The Babraham Institute	https://github.com/FelixKrueger/TrimGalore
Fastx Toolkit 0.7	Hannon Lab	http://hannonlab.cshl.edu/fastx_toolkit/index.html
CD-HIT 4.6	(Fu et al., 2012)	http://cd-hit.org/
R 3.6.2	N/A	https://www.r-project.org/
IDBA-UD assembler 1.1.1	(Peng et al., 2012)	https://i.cs.hku.hk/∼alse/hkubrg/projects/idba_ud/
Bowtie 2.3.5	(Langmead and Salzberg, 2012)	http://bowtie-bio.sourceforge.net/bowtie2/index.shtml
VirSorter 2.2.1	(Guo et al., 2021)	https://github.com/jiarong/VirSorter2
QIIME 1.9	(Caporaso et al., 2010)	http://qiime.org/
DESeq2 3.13	(Love et al., 2014)	https://bioconductor.org/packages/release/bioc/html/DESeq2.html
Frag Gene Scan 1.31	(Rho et al., 2010)	https://sourceforge.net/projects/fraggenescan/
Meta Genome Analyzer 6.18.3	(Huson et al., 2018)	https://software-ab.informatik.uni-tuebingen.de/download/megan6/welcome.html

**Other**		

In-house scripts describing the data analysis process	This paper	https://github.com/lab8a/2021-iScience-Phageome

### Resource availability

#### Lead contact

Further information and requests for resources and reagents should be directed to and will be fulfilled by the lead contact, Adrian Ochoa-Leyva (adrian.ochoa@ibt.unam.mx).

#### Materials availability

This study did not generate new unique reagents.

#### Data and code availability

All original code describing the data analysis process are available on GitHub at https://github.com/lab8a/2021-iScience-Phageome. The sequence data have been deposited in the NCBI under the NCBI BioProject accession number: PRJNA646512. Accession numbers are also listed in the key resources table. Any additional information required to reanalyze the data reported in this paper is available from the lead contact upon request.

### Method details

#### Experimental model and subject details

The information on the fecal samples and the biochemical parameters information used in this study are fully described in a previous study of our group(Gallardo-Becerra et al., 2020).Briefly, we analyzed the stools from 10 normal weight (NW), 10 obese (O), and 8 obese with metabolic syndrome (OMS) children, aged 7–10 years old. All children came from households with a middle economic class income and belonged to a similar socio-cultural status. All of them lived in Mexico City at the time of collection and did not practice any sport regularly.

The study groups were paired by gender and age (Table S1). Feces samples were collected and refrigerated at 4°C and transported to the research facility within the following 12 h in a portable cooler with ice packs to preserve the temperature. At the research facility samples were aliquoted into 200 mg portions in sterile plastic containers with RNA later and stored at −70°C. For the biochemical parameters, 5 mL of blood samples were drawn after 8–12 h fasting on the same day of the feces collection. Also, anthropometric parameters, blood pressure, and body mass index were measured following standardized procedures, as previously described(Gallardo-Becerra et al., 2020).

Obesity was defined by a body mass index (BMI) ≥ 95th percentile. In contrast, NW was defined as a BMI between the 15th and 75th percentiles considering age and gender, based on the Centers for Disease Control and Prevention (CDC). Metabolic syndrome parameters were determined according to previous reports(De Ferranti et al., 2004), and OMS were defined by the presence of waist circumference >75th percentile considering age and gender, and at least two of the following metabolic traits: (1) triglycerides > 1.1 mmol/L (100 mg/dL); (2) HDL cholesterol < 1.3 mmol/L (50 mg/dL), (3) glucose > 6.1 mmol/L (110 mg/dL) and (4) systolic blood pressure > 90th percentile considering gender, age, and height. Children in the O group were selected with no more than one metabolic syndrome trait.

Exclusion criteria for all samples included recent bodyweight loss > 10%, antibiotic intake 3 months before sample collection, and the occurrence of diarrhea or acute gastrointestinal illness during the same period. The Ethics Committee of the Instituto Nacional de Medicina Genómica (INMEGEN) in Mexico City, Mexico, approved the study. Each child's parents or legal guardians signed the informed consent form for participation, and all children assented to participate.

#### Viral-like particles (VLPs) isolation

Viral-like particles (VLPs) were isolated from ∼250 mg of fecal sample suspended by vortexing in 1 mL of sterile SM Buffer (pH 7.5), (Cat. 725-2520, Nalgene, NY, USA) to stabilize phage particles. The homogenates were centrifuged at 4,700 × g for 30 min at room temperature, and the supernatant was filtered through 0.45 μm (Cat. 725-2545, Nalgene) and 0.22 μm PES filters (Cat. 725-2520, Nalgene NY, USA) to remove cell debris and bacterial-sized particles. The filtrate was then re-suspended in 15 mL of SM buffer and concentrated to 200 μl at 4°C with an Amicon Ultra 15 filter unit, 100 KDa (Cat. UFC910024, Millipore, MA, USA) to remove cellular debris. The concentrate was transferred to a 1.5 mL microfuge tube and incubated with 40 μl chloroform for 10 min at room temperature to degrade any remaining bacterial and human cell debris. Non-virus protected DNA was eliminated with 2.5 units per milliliter of DNase I following the manufacture's procedure (Cat 18047-019, Invitrogen, MA, USA). After incubation, the DNase was inactivated at 65°C for 10 min. The samples were stored at -80°C until further processing.

#### Microscopy visualization and VLPs counts

We used epifluorescence microscopy to quantify the isolated VLPs. We stained 10 μl of the concentrated VLPs samples with a mix of SYBR Green (Cat. K0221, Thermo Fisher Scientific, MA, USA) and 10 ul of paraformaldehyde previously filtered through 0.22 μm PES membranes (Cat. 725-2520, Nalgene NY, USA). Five fields per sample were observed and quantified with an Olympus FV1000 Multiphoton Confocal Microscope, and each field was quantified in triplicate using the free image processing software Fiji. The average number of VLPs from the five fields by triplicate was used per sample to calculate the amount per gram of feces per sample (Table S3). Additionally, an aliquot of 8 μl from the concentrated VLPs samples was observed in the transmission electron microscope to corroborate the phage nature (morphology and structure) in VLPs (Figure 1B).

#### Viral DNA shotgun sequencing

The DNA of VLPs was extracted following the manufacture's protocol for the QIAamp MinElute Virus Spin Kit (Cat. 57704, Qiagen, Hilden, Alemania). The resulting DNA for each sample was quantified with a Qubit fluorometer (Cat. Q32851, Thermo Fisher Scientific, CA, USA), and diluted with ribonuclease-free water to a concentration of 0.3 ng/μl. From this DNA, we prepared independent sequencing libraries following Illumina Nextera XT DNA Library Preparation protocol (Cat. FC-131-1024, Illumina, CA, USA) that supports ultra-low DNA input with unique barcodes for multiplexing. For DNA tagmentation, we mixed 5 μl of DNA at 0.3 ng/μl with the tagmentation reaction mix. Next, we added the indexed oligos and amplified the library for 12 cycles. Each library was purified with 30 μl of AMPure XP beads (Cat. A63881, Beckman Coulter, CA, USA) to obtain ∼600 bp DNA fragments.

The size and quality of each library were assessed with a DNA bioanalyzer 2100 (Cat. 5067-4626, Agilent Technologies, CA, USA). All barcoded libraries were pooled together and sequenced using the Illumina NextSeq500 platform in the 2x150 pair-end mode at the Sequencing Unit Facility of the National Institute for Genomic Medicine, México.

#### Cleaning and clustering of sequenced reads

Total reads were dereplicated, adapters and low-quality bases (PHRED Q30) were trimmed using Trim_Galore (https://github.com/FelixKrueger/TrimGalore), and the first 20 nucleotides were removed with Fastx Toolkit (http://hannonlab.cshl.edu/fastx_toolkit/index.html). Human and bacterial reads by read mapping using BWA (against the Homo sapiens GRCh38.p13 reference genome GenBank GCA_000001405.28) and Kraken (Wood and Salzberg, 2014) against bacteria NR database, with default parameters. All reads mapped to those genomes were removed, and the remaining reads were named quality-filtered reads. Quality-filtered reads were clustered at a 95% identity using CD-HIT (Fu et al., 2012) to remove redundancy and generate a unique sequence dataset.

#### Analysis of viral reads richness

The viral richness between groups (NW, O, OMS) was determined, collecting 1,000 random subsamples of 149,000 single-end quality-filtered reads using seqtk subseq according to the smallest sample (NW_169: 149,775), and later each sub-sample was clustered at a 95% identity level using CD-HIT to identify the unique groups of reads.

#### Functional profiles and pVOGs analysis

The quality-filtered reads were mapped onto the viral NR RefSeq and POGs databases using BLASTX with a maximum e-value cutoff of 0.001 and a maximum of 50 reported target sequences. After mapping, an abundance matrix was generated using an in-house bash script. The matrix was then annotated according to the KEGG annotation of each protein using the UniProtKB online database and an in-house bash script. The KEGG functional profile was then generated using the relative abundance for each protein and function for each sample. The quality-reads were mapped against the Prokaryotic Virus Orthologous Groups (pVOGs) database using BLASTX with a maximum e-value cutoff of 0.001, and a maximum of 50 reported target sequences. The final results were filtered with an in-house bash script to get the final results with the pVOGS classification for each sample.

#### Classification of viral reads

The quality-filtered unique sequences were taxonomically classified into orders and families, according to the International Committee on Taxonomy of Viruses (ICTV), using BLASTX with maximum e-value cutoff 0.001against the NR RefSeq viral database and considering the lowest-common ancestor algorithm in MetaGenomeAnalyzer (MEGAN6) (Huson et al., 2018) using the following parameters: Min Support: 1, Min Score: 40.0, Max Expected: 0.01, Top Percent: 10.0, Min-Complexity filter: 0.44. Absolute read counts for selected viral taxa were normalized using all the reads from each sample, obtaining the relative abundance for each sample using R scripts.

#### De Novo contig assembly

The total quality-filtered reads from all samples were pooled for *de novo* assembly using IDBA-UD assembler (Peng et al., 2012) with a k-mer length of 20-125 with scaffolding rounds. Each sample's reads were mapped separately with Bowtie2 (v2.3.5) (Langmead and Salzberg, 2012) against the viral assembly using the end-to-end mode with the default parameters. Viral scaffolds covered ≥80% in length by the reads by at least one sample were used as a cut-off to discard chimeras. Next, we kept the scaffolds ≥ 4 Kb for downstream analysis to reduce the probability of selecting viral genome fragments.

To eliminate the redundant contigs, we used CD-HIT using a 95% clustering identity. To know the number of genes per contig length, we conducted the gene prediction of each contig using FragGeneScan (Rho et al., 2010) with the following parameters -complete=0 -train=illumina_5.

#### Taxonomic classification of *de novo* assembly

The taxonomy classification of each contig was obtained using dc_megablast against the NT NCBI viral genomes database with maximum e-value cutoff 0.001 and the maximum number of target sequences to report set to 50 hits (Moreno-Gallego et al., 2019). The taxonomy of each contig was assigned by the lowest-common-ancestor algorithm in MEtaGenomeANalyzer (MEGAN6) using the discontinuous megablast results with the following parameters: Min Support: 1, Min Score: 40.0, Max Expected: 0.01, Top Percent: 10.0, Min-Complexity filter: 0.44. After that, all contigs without taxonomic classification with dc_megablast were searched against the NR NCBI viral protein database using BLASTX with the maximum e-value cutoff 0.001 and a maximum number of reported target sequences set to 50. The taxonomy of each contig was assigned by the lowest-common- ancestor algorithm in MetaGenomeANalyzer (MEGAN6), using the BLASTX results with the following parameters: Min Support: 1, Min Score: 40.0, Max Expected: 0.01, Top Percent: 10.0, Min-Complexity filter: 0.44. The assembly of 12,287 contigs was classified using VirSorter2 (v2.2.1) (Guo et al., 2021) with the default parameters following the tutorial provided by the authors. After that, we contrasted the VirSorter classification against the previous one using the NT NCBI viral genomes and NR NCBI viral protein databases (Guo et al., 2021). The assembly of 12,287 contigs was mapped against the Prokaryotic Virus Orthologous Groups (pVOGs) database using BLASTX with a maximum e-value cutoff 0.001, and maximum target sequences to report set to 50.

#### Differential abundance of phage contigs

The recruitment of reads to the contigs assembly was used to construct an abundance matrix, applying the filter of coverage and length as previously recommended(Roux et al., 2017). The coverage was defined from reads mapping (Bowtie2) at ≥90% identity and ≥80% length. Mapping outputs were converted into an abundance matrix using an in-house R script normalized by Reads Per Kilobase per Million sequenced reads per sample (RPKM)(Reyes et al., 2015).

#### Richness and diversity of phage contigs

Richness and diversity of the contig assembly were evaluated based on the median of 10,000 rarefactions at a depth of the smallest sample based on the RPKM matrix using QIIME 1.9 (Caporaso et al., 2010). Based on the presence of phage contigs in the samples, each phage contig was classified as either core phages: detected in >80% of the samples; common phages: in >50% and <80%; and individual phages: appearing in <50% of the population.

#### Bacteria and biochemical parameters correlations

All correlations were calculated using the Spearman coefficient with rcorr function in R considering all samples. We considered the RPKM matrix for the contigs phage abundance and the relative frequency of the significant over-abundant taxa previously reported between NW, O, and OMS for the microbiota abundance(Gallardo-Becerra et al., 2020).

### Quantification and statistical analysis

The differences in the number of VLPs between the three groups (Figure 1C) were evaluated with pairwise Mann–Whitney–Wilcoxon non-parametric tests in R.

For each sample of the analysis of viral reads richness (Figure S4), the median of all unique observations was calculated. The resulting groups were tested for normality with Shapiro-Wilk tests in R. Differences were evaluated with pairwise Mann–Whitney–Wilcoxon non-parametric tests in R.

The RPKM matrix was used to determine statistical differential viral taxonomic abundances between groups using a Kruskall Wallis test using the Holm-Sidak method, with alpha= 0.01 for multiple test correction (Figure 2). This matrix was also used to determine statistical differential individual contig-abundance (Figures 4A and 4B) using DESeq2 (Love et al., 2014) with log fold change ≥2 and FDR adjustment (p-value≤ 0.05) using Benjamini-Hochberg correction.

Medians for richness and diversity of phage contigs were obtained per sample and groups were evaluated for normality, following pairwise Mann–Whitney–Wilcoxon non-parametric tests in R (Figures 3A and 3B).

The prevalence of highly abundant contigs found in >80% of samples in the NW group was compared across the three groups using pairwise Mann–Whitney–Wilcoxon non-parametric tests using R (Figure S14).

For the bacteria and biochemical and anthropometric correlations, we selected the Spearman correlations with R2 >0.3 and p-value ≤0.05 (Figures 6, S13, S15, S17, and S18). However, after applying an FDR correction for the p-values, we did not get a significant correlation below the 0.05 cutoff. Thus, to still attend to the tendencies among groups, we referred to the unadjusted p-values in the correlation analysis.

For the Euclidean distances comparison the relative abundance phage tables were subjected to a center log-ratio transformation with the mixOmics library v6.10.9 in R using an offset of min_value 1e-7 to deal with zero logs. The resulting Euclidean matrix was then subjected to dimensional reduction with a principal components analysis (with vegan 2.5-6 in R). Euclidean distances were then calculated to create an adjacency matrix for group testing with ANOSIM and adonis (carried out with vegan) and posthoc pairwise group testing (Figure S12).

## Supplemental information

Document S1. Figures S1–S18 and Tables S1–S3, S5, and S6

Table S4. Read sequences assignment per sample, related to Figures S1 and S4 and to STAR Methods

Table S7. Relative abundance of the taxonomic classification of 4,611 phage contigs per sample, related to Figure S10 and STAR Methods
