# Supplementary material for: Gut dsDNA virome shows diversity and richness alterations associated with childhood obesity and metabolic syndrome
Source: iScience. 2021 Jul 24;24(8):102900. doi: 10.1016/j.isci.2021.102900 (PMC8361208; doi:10.1016/j.isci.2021.102900)
Supplement: Document S1. Figures S1–S18 and Tables S1–S3, S5, and S6 [file mmc1.pdf]

iScience, Volume *24*

Supplemental information

Gut dsDNA virome shows diversity and richness alterations associated with childhood obesity and metabolic syndrome

Shirley Bikel, Gamaliel López-Leal, Fernanda Cornejo-Granados, Luigui Gallardo-Becerra, Rodrigo García-López, Filiberto Sánchez, Edgar Equihua-Medina, Juan Pablo Ochoa-Romo, Blanca Estela López-Contreras, Samuel Canizales-Quinteros, Abigail Hernández-Reyna, Alfredo Mendoza-Vargas, and Adrian Ochoa-Leyva

Supplemental information

Gut dsDNA Virome Shows Diversity and Richness Alterations Associated to Childhood Obesity and Metabolic Syndrome

Shirley Bikel, Gamaliel López-Leal, Fernanda Cornejo-Granados, Luigui Gallardo-Becerra, Rodrigo García-López, Filiberto Sánchez, Edgar Equihua-Medina, Juan Pablo Ochoa-Romo, Blanca Estela López-Contreras, Samuel Canizales-Quinteros, Abigail Hernández-Reyna, Alfredo Mendoza-Vargas and Adrian Ochoa-Leyva

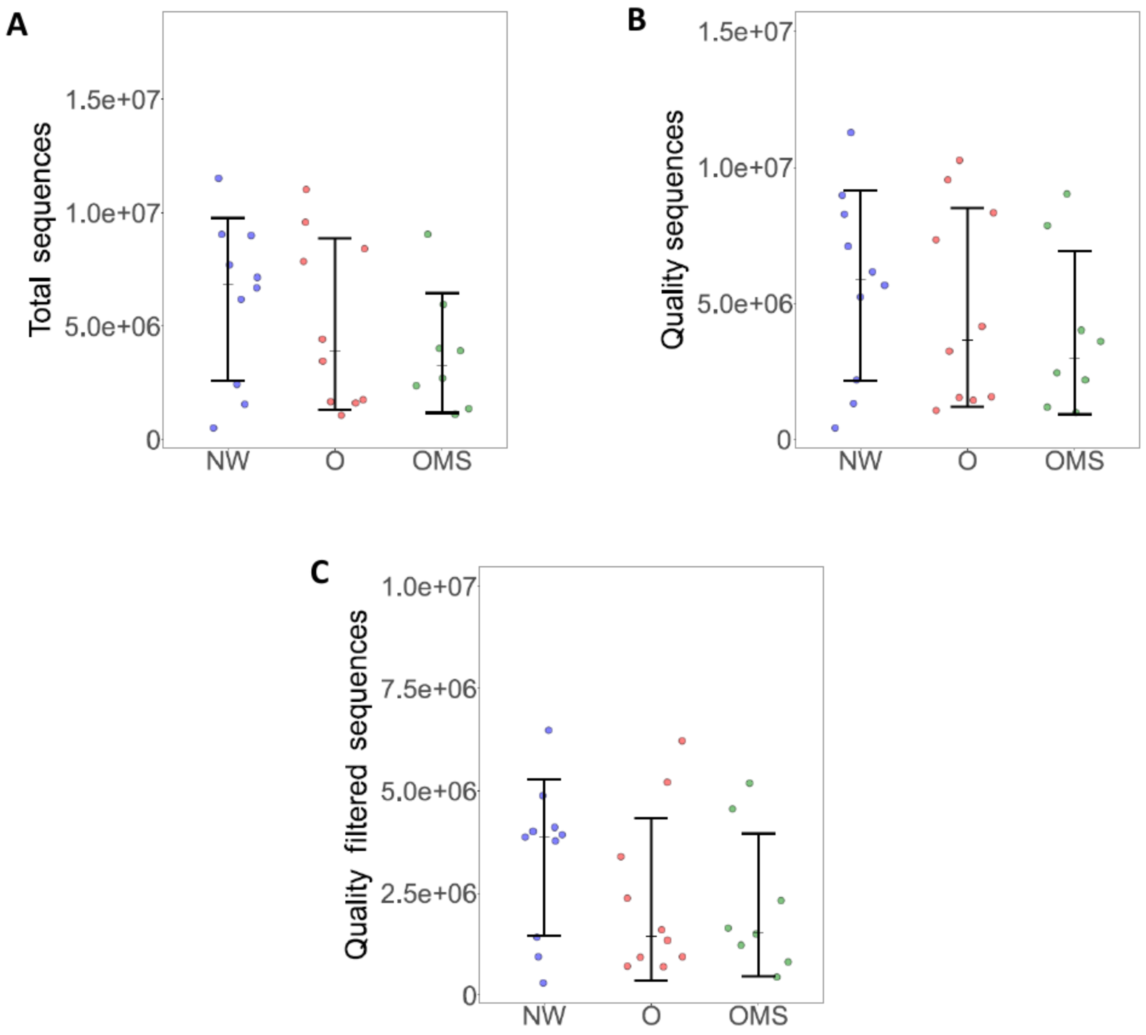

Figure S1. VLPs sequences detected in NW, O, and OMS samples, Related to Table S4 and to STAR Methods. Plots containing the total number of sequences (A), quality-filtered sequences (B), and quality-filtered sequences (human and bacteria sequences were removed) (C) are showed. Error bars indicate the median and interquartile range. The number of sequences per sample is shown as points. There were no statistically significant differences among groups.

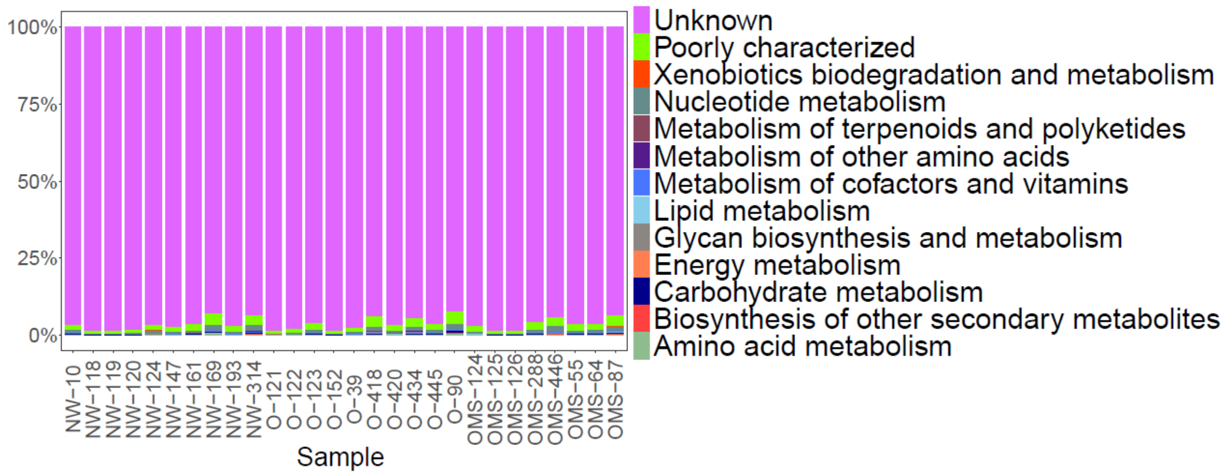

Figure S2. Functional assignment of VLPs, Related to Table S4 and to STAR Methods. The relative abundance of KEGG categories in VLPs derived reads. "Unknown" (purple) indicates the proportion of reads that cannot be functional classified.

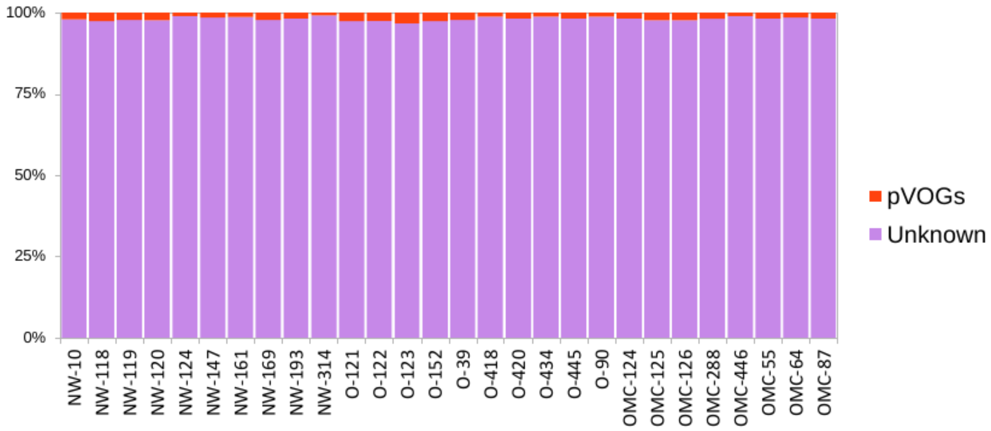

Figure S3. The relative abundance of pVOGs categories in VLPs derived reads, Related to Table S4 and to STAR Methods. "Unknown" (purple) indicates the proportion of reads that cannot be assigned to pVOG categories.

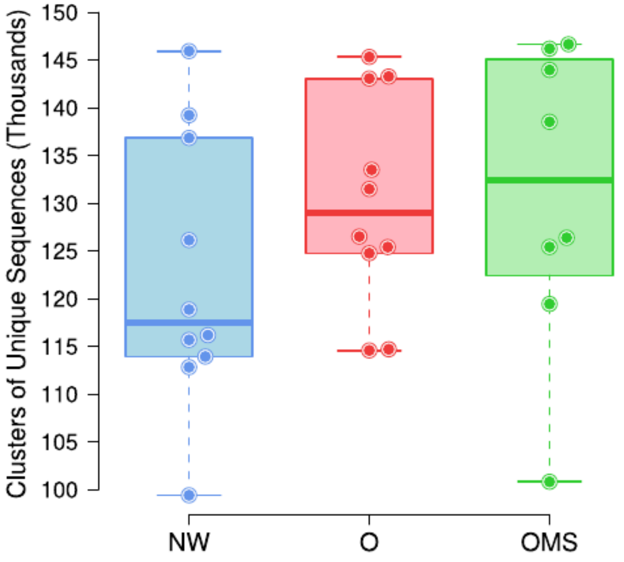

Figure S4. Total unique clusters (y-axis) per group, Related to Table S4 and to STAR Methods. Each point shows the median of 1,000 iterations at a sequence depth of 149,000 reads for each sample. The boxes show the distribution for each group. Error bars indicate the median and interquartile range.

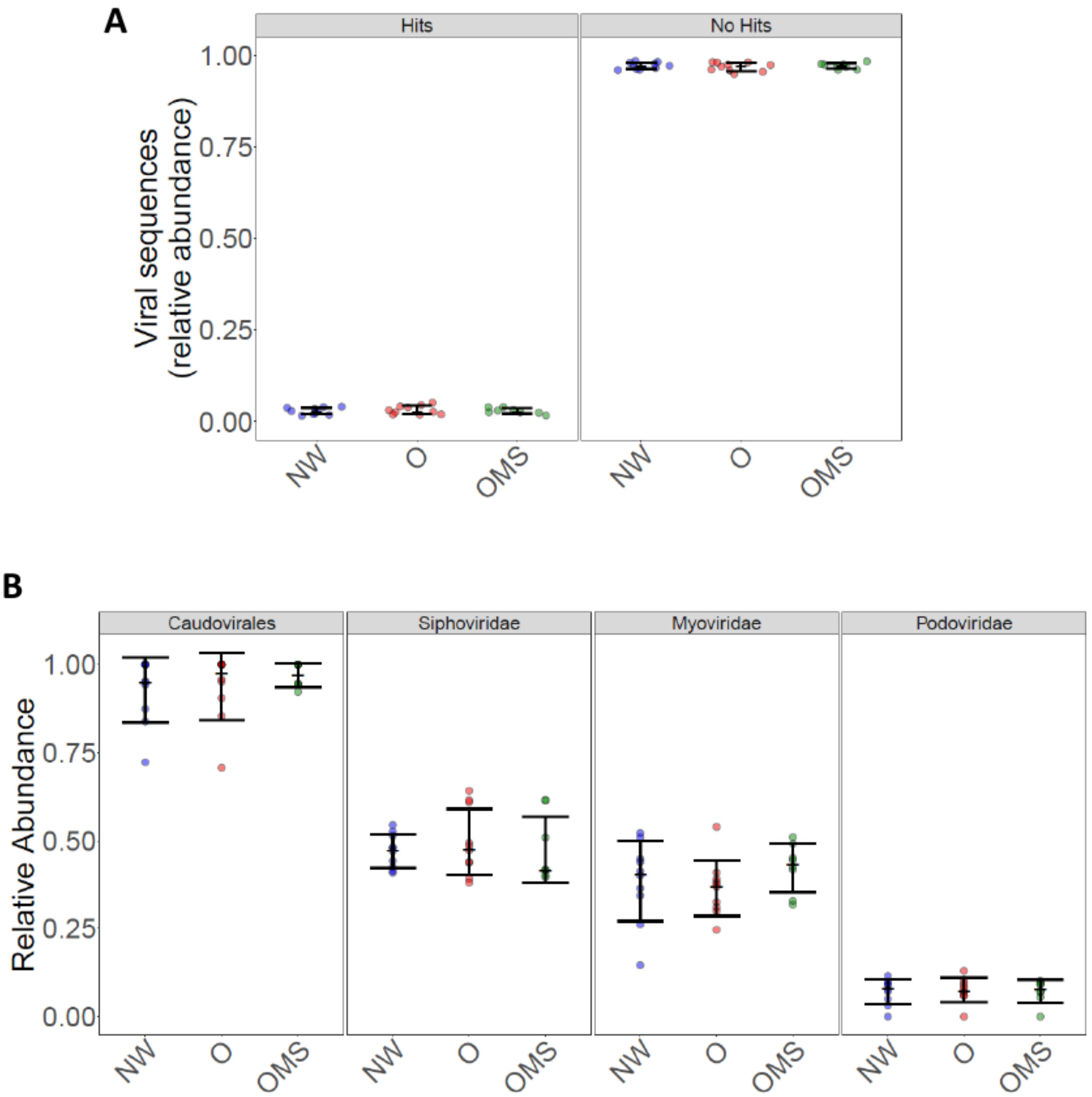

Figure S5. Viral taxonomic assignment of VLPs derived reads, Related to Table S4 and to STAR Methods. A. The relative abundance of unique sequences matched against a viral protein sequence (hits), or not matched (no hits), is shown for each group. **B.** The relative abundance of unique sequences assigned to Caudovirales and their taxonomic family members in NW, O, and OMS groups. The number of unique sequences per sample is shown as points. Error bars indicate the median and interquartile range.

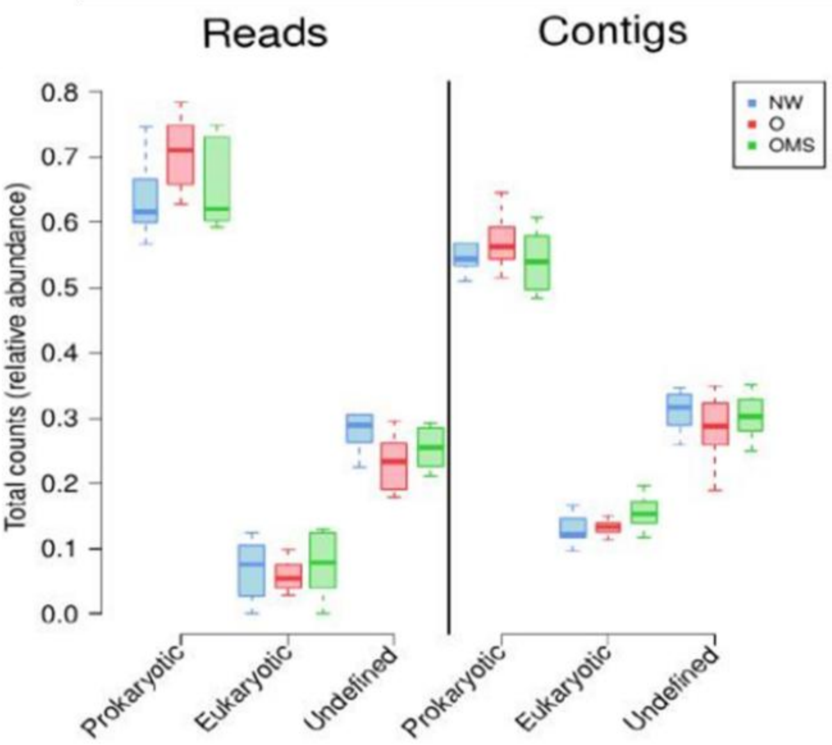

Figure S6. Taxonomic classification of viral reads and contigs, Related to Table S4 and to STAR Methods. The relative abundance of sequencing reads and contigs were assigned to the indicated viral classification. Error bars indicate the median and interquartile range.

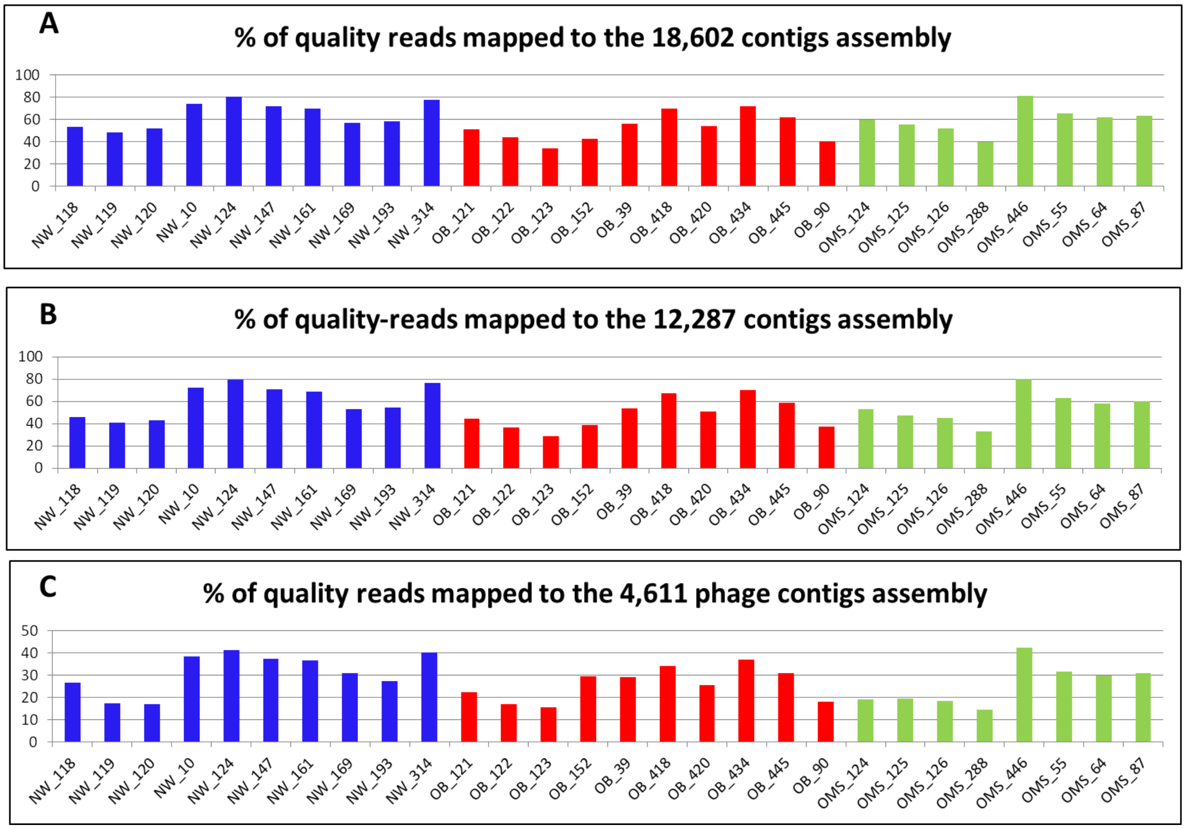

Figure S7. Percentage of quality-reads mapped back to the different contig assemblies, Related to Table S6 and to STAR Methods. A) Assembly of the 18,602 contigs (whole virome assembly), B) Assembly of the 12,287 contigs >4Kb, C) Assembly of the 4,611 contigs classified as phages.

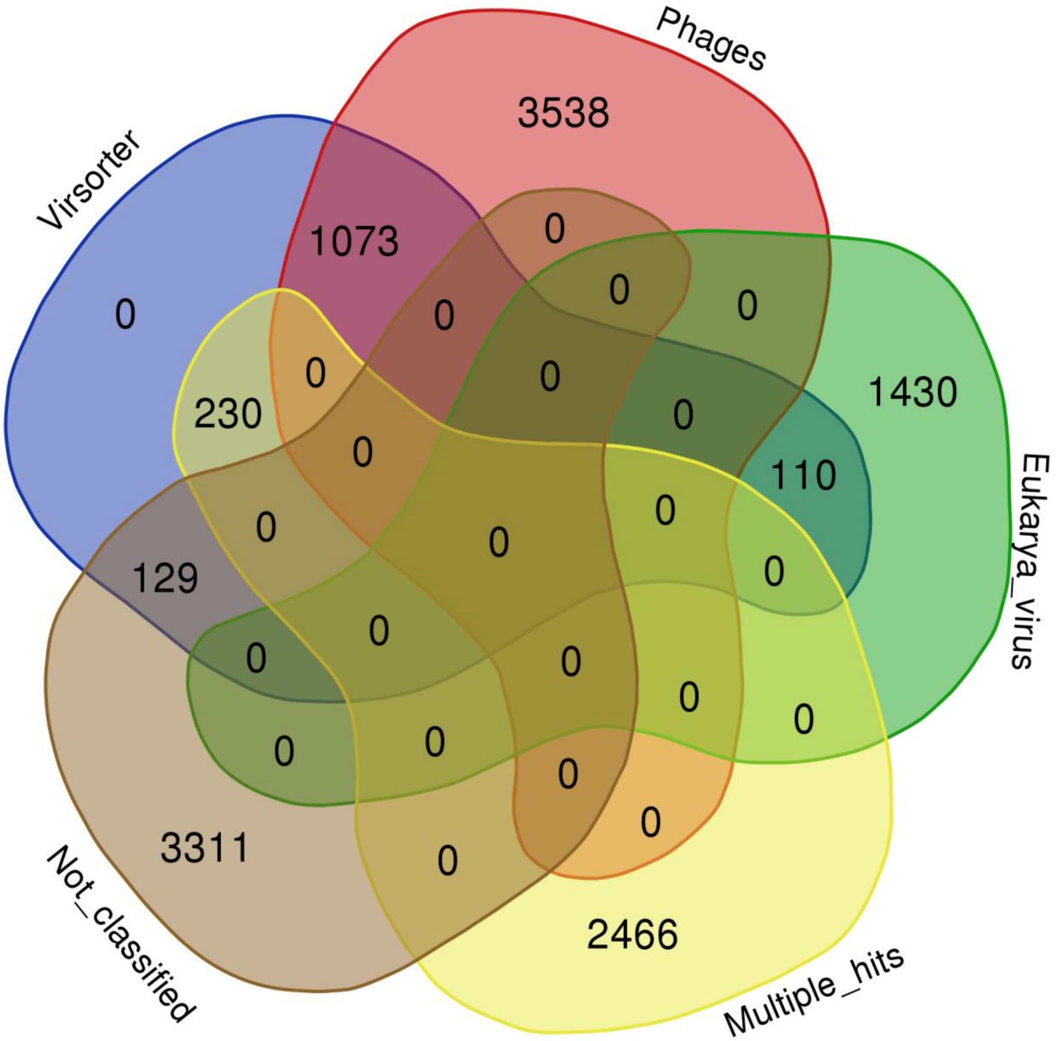

Figure S8. Venn diagram showing the overlap between the three classifications obtained from NR and NT and the contigs classified using Virsorter, Related to Table S7 and to STAR methods.

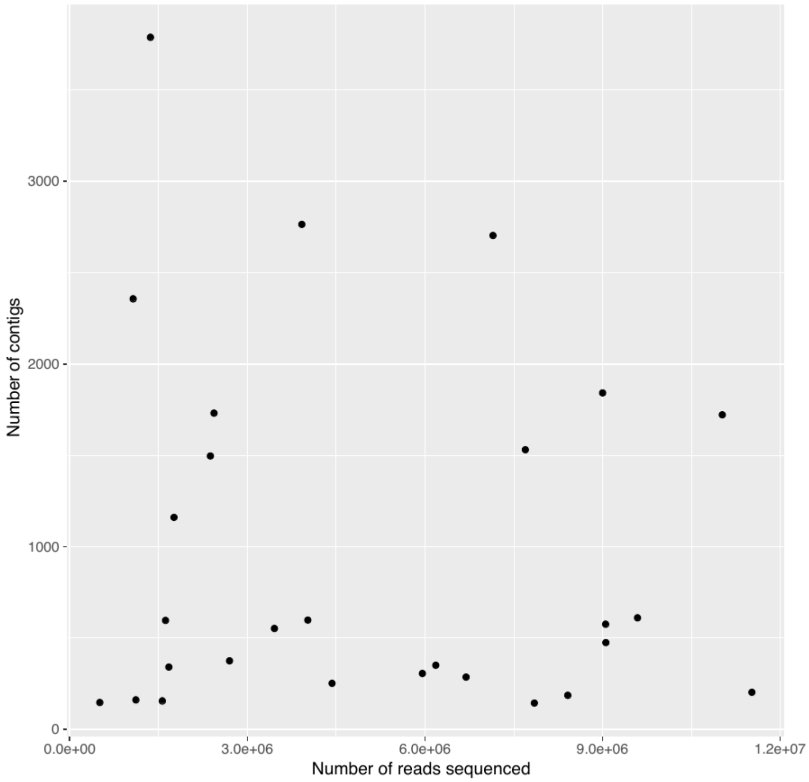

Figure S9. Correlation between quality reads and the number of contigs per sample. R^2 = 0.0131, p-value= 1, **Related to Tables S4 and S6 and to STAR methods.**

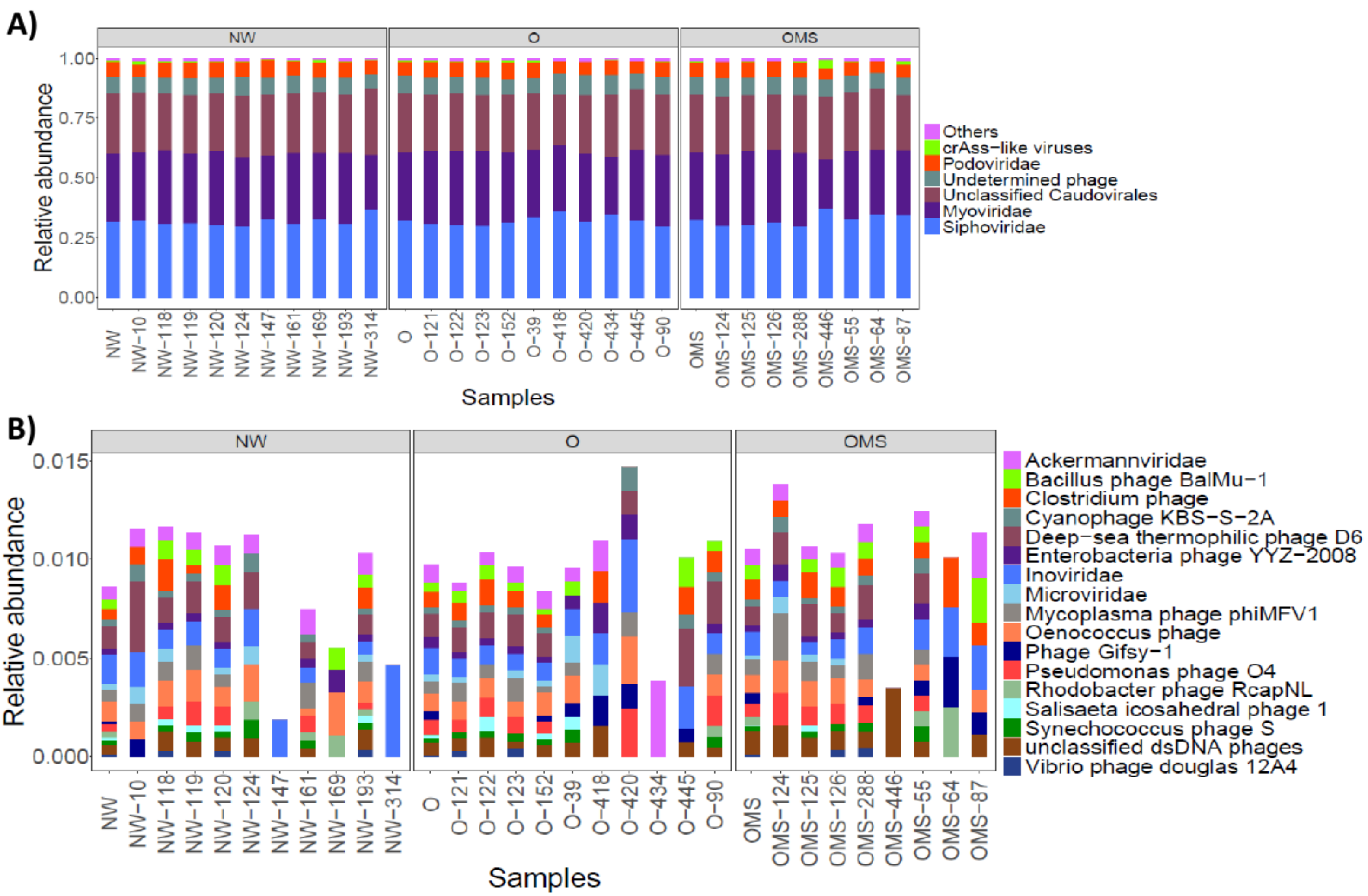

Figure S10. Relative abundance of phage contigs per sample and group, Related to Table S7 to STAR methods. A) Relative abundance of classified phage contigs. B) Relative abundance of the less abundant phage contigs assigned to "Others" in A.

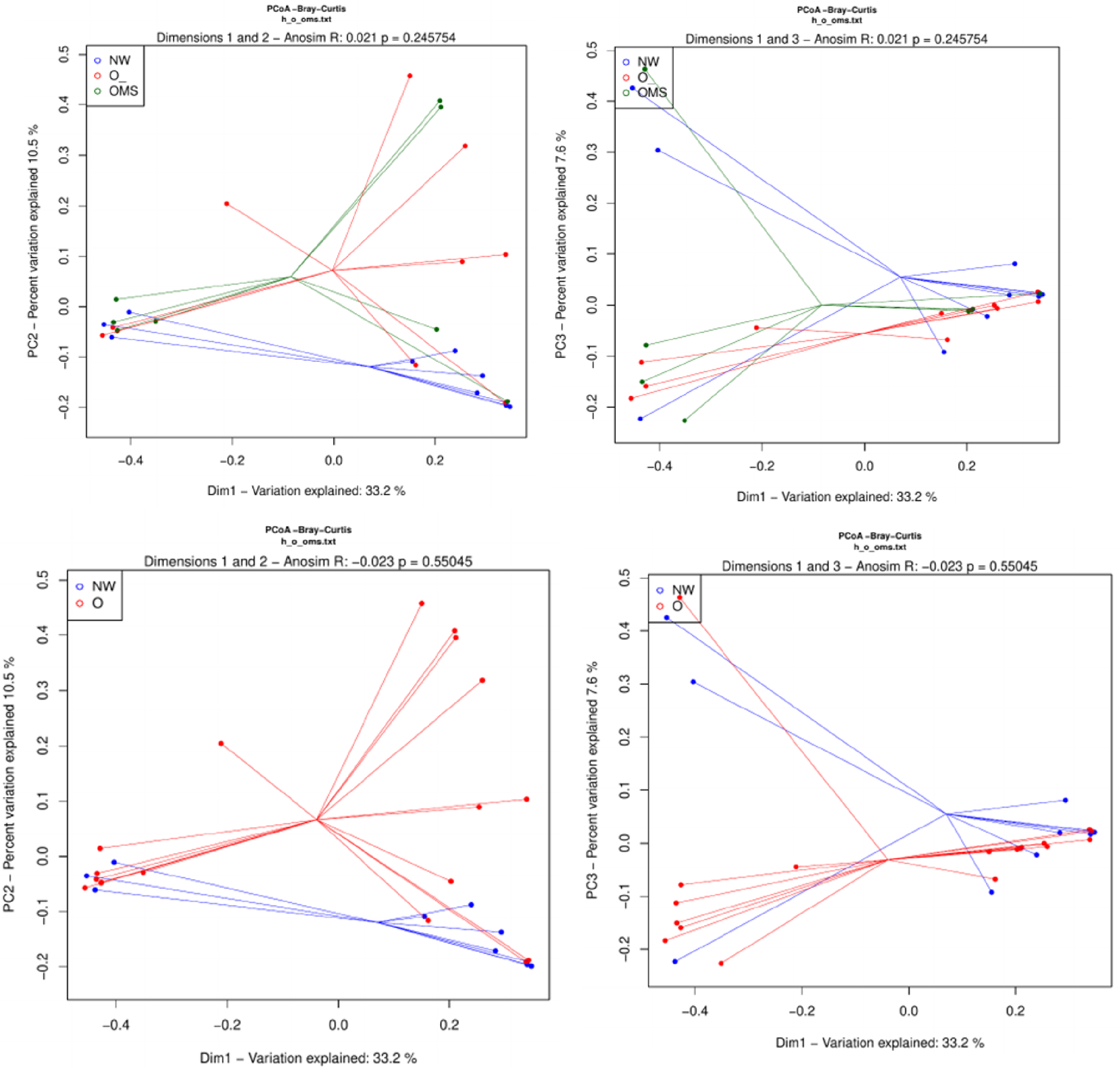

Figure S11. Principal Coordinates Analysis (PCoA) based on Bray-Curtis dissimilarity, Related to figure 3C and D. The samples were tagged as NW, O, and OMS: A) PC1 vs PC2 and B) PC1 vs PC3. PCoA based on Bray-Curtis dissimilarity with samples tagged by all obese (O + OMS) and NW: C) PC1 vs PC2 and D) PC1 vs PC3.

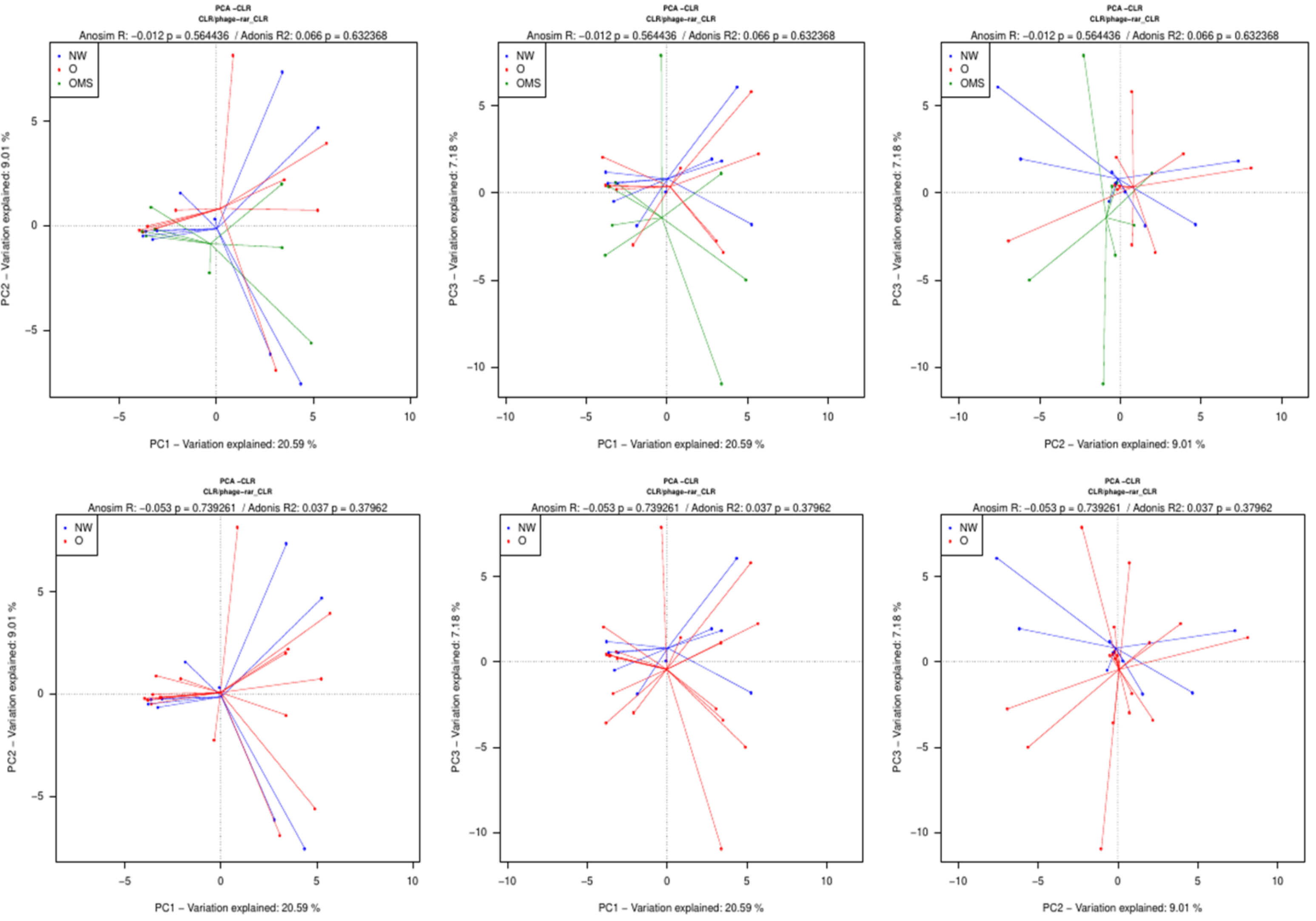

Figure S12. PCA based on CLR coordinates, Related to figure 3C and D and to STAR methods. Samples were tagged as NW, O and OMS: A) PC1 vs PC2: B) PC1 vs PC3; and C) PC2 vs PC3. Samples tagged by all obese (O + OMS) and NW: D) PC1 vs PC2: E) PC1 vs PC3; and F) PC2 vs PC3.

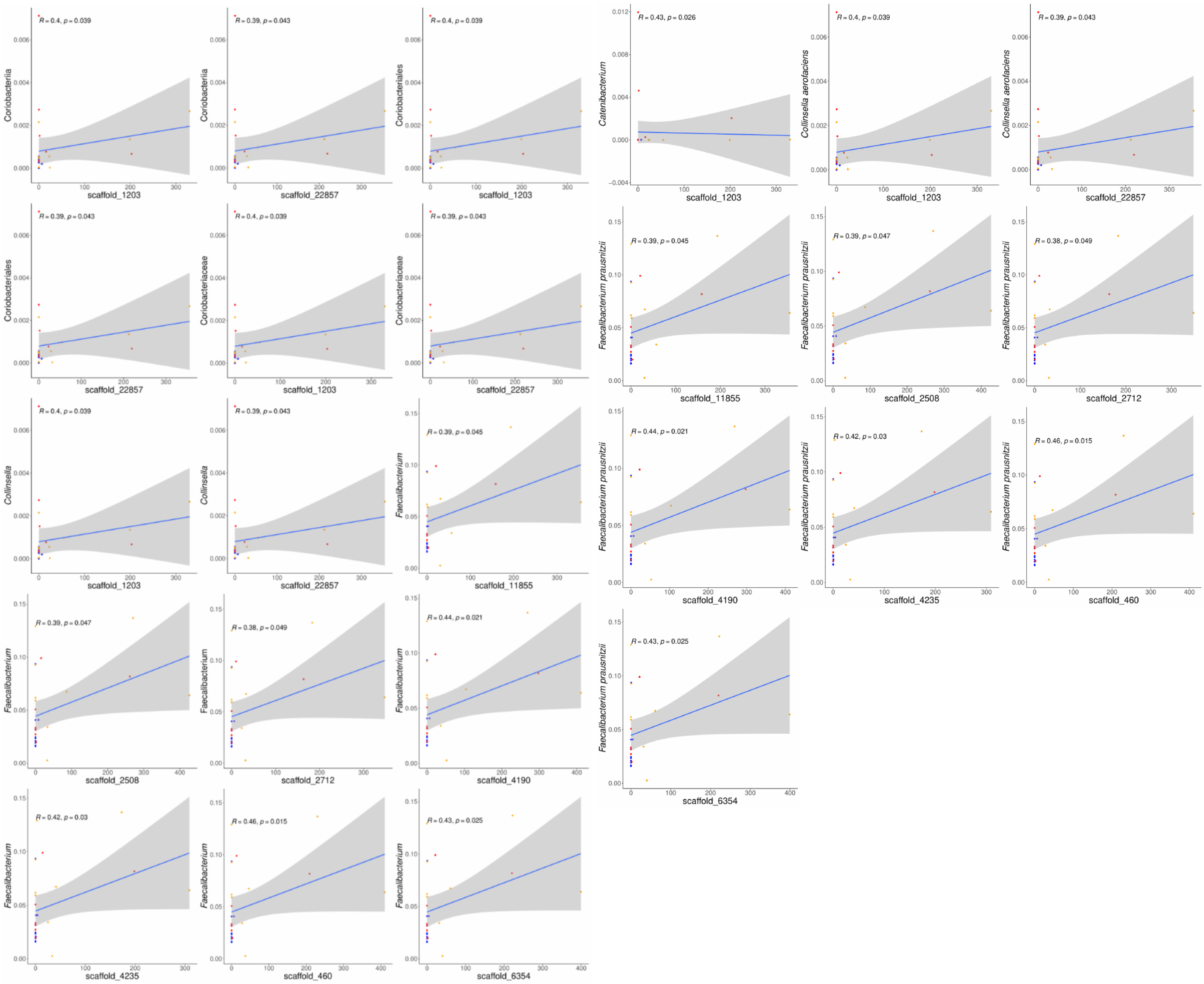

Figure S13. Spearman correlation plots, Related to figure 4D and to STAR methods. Spearman correlation between bacterial taxa altered in obesity and metabolic syndrome and the abundance of the 48 overabundant phage contigs shared in both O and OMS samples. Blue circles = NW samples; orange circles = O samples; and red circles = OMS samples.

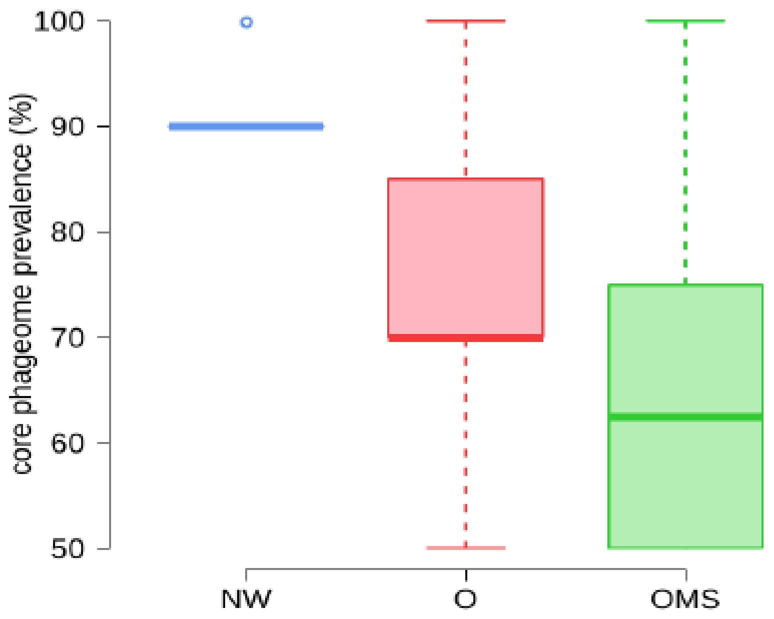

Figure S14. Prevalence of phage contigs with a higher presence (>80% of samples) in NW with respect to O (p-value= <0.0001) and OMS (p-value= <0.0001) samples, Related to the STAR methods. Error bars indicate the median and interquartile range.

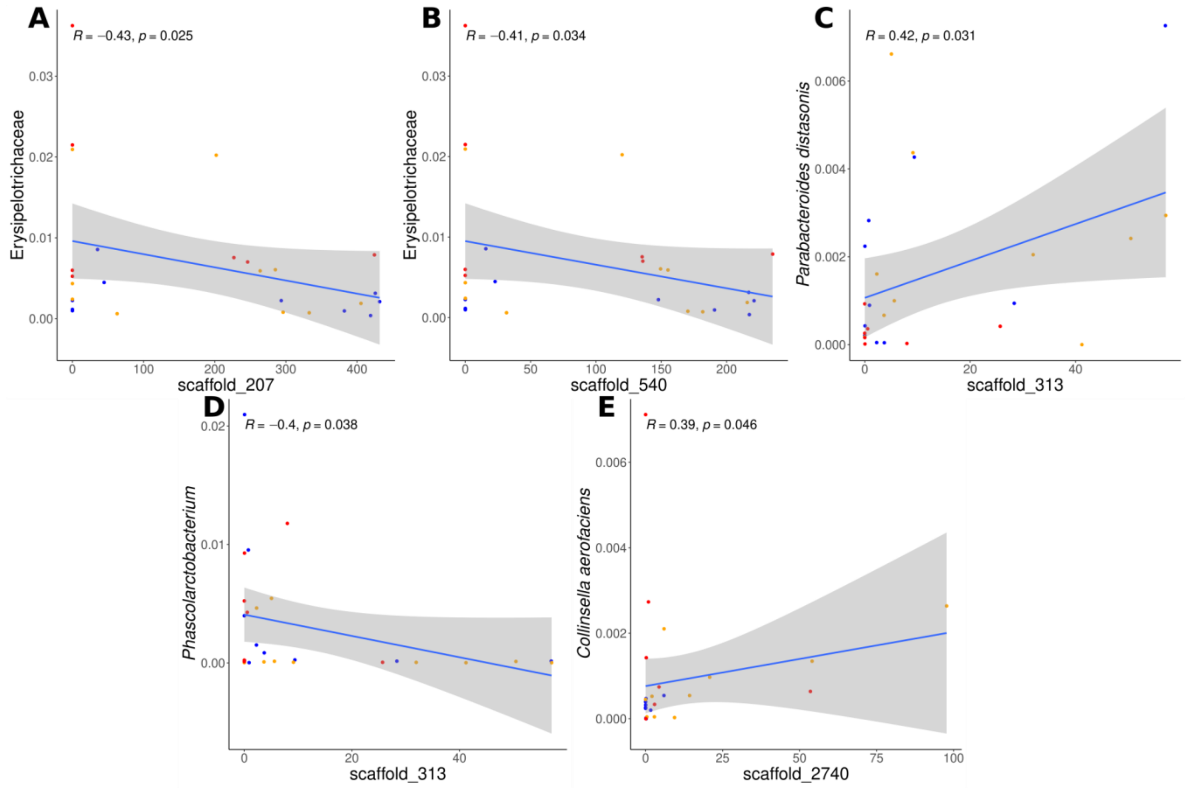

Figure S15. Spearman correlation plots of the contigs that significantly correlated with disease specific bacteria, Related to Figure 6A. The y-axis shows the abundance of the microbial taxa, and the x-axis shows the abundance in RPKM for each contig. Blue circles = NW samples; orange circles = O samples; and red circles = OMS samples.

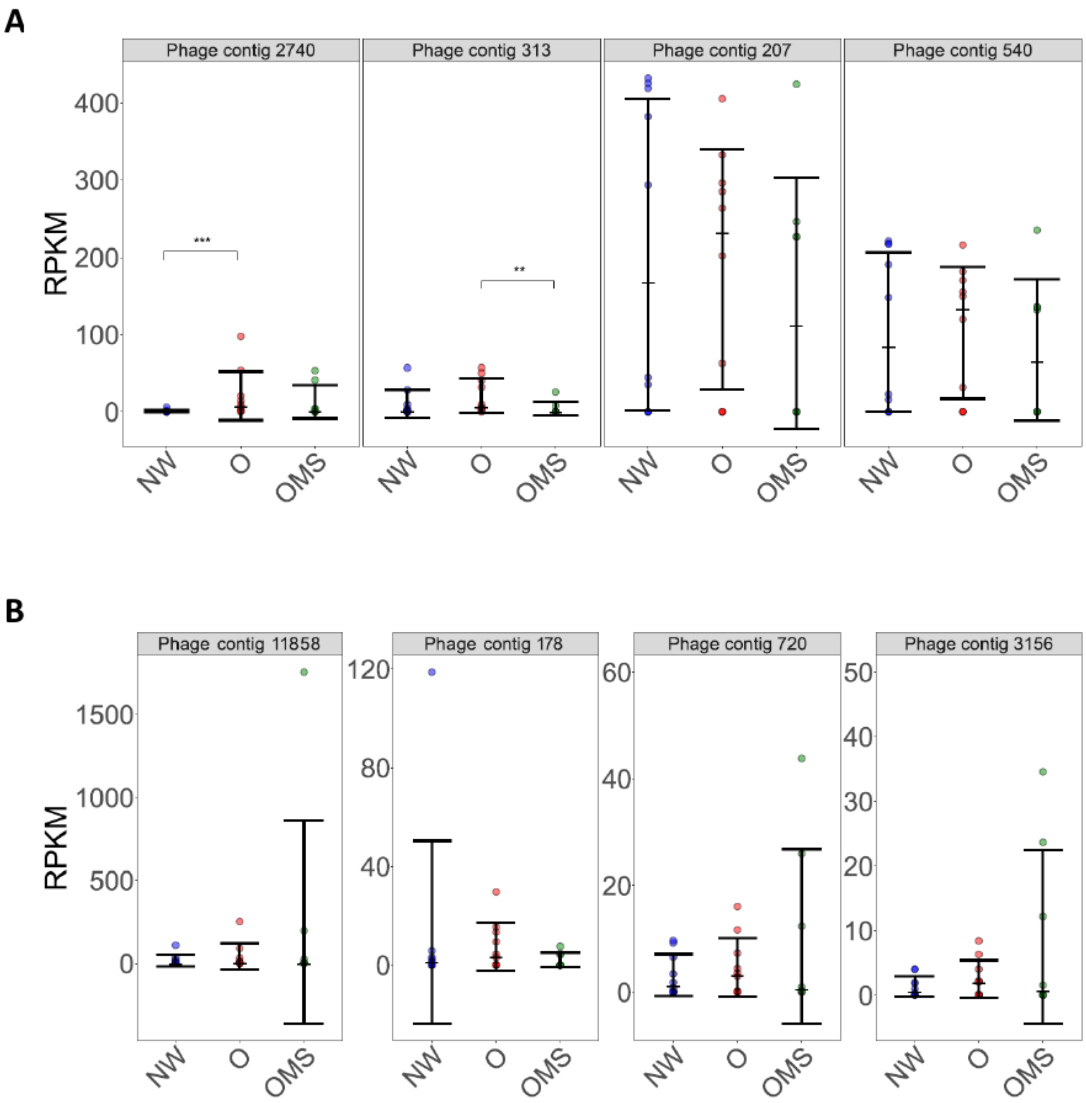

Figure S16. Box plots of the phage contig abundances, Related to Figure 6. The phage contigs in NW, O, and OMS groups that significantly correlated with **A:** bacterial taxa altered by obesity and obesity with metabolic syndrome and **B:** the clinical and anthropometrical parameters of obesity and metabolic syndrome. The phage contigs 2740, 313, and 207 showed in **"A"** also correlated with clinical and anthropometrical parameters. Points represent the phage contig abundances (RPKM) per sample. Error bars indicate the median and interquartile range per group.

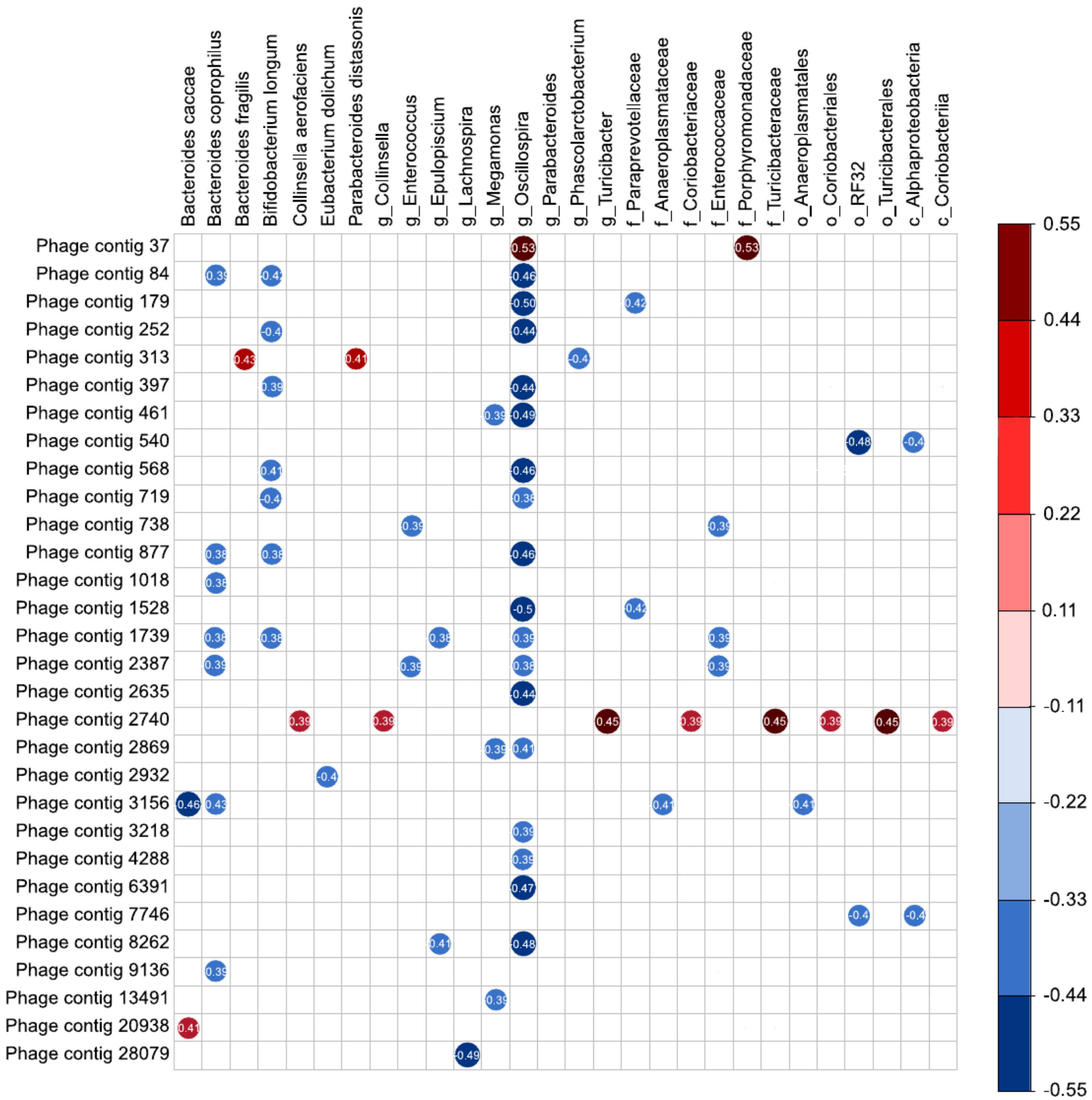

Figure S17. Core phage contigs-bacteria correlation plots, Related to figure 5A. Spearman correlation plots of the phage abundances (RPKM) of the 48 phage contigs with a higher prevalence (≥80% of all the samples) and the relative abundance of all the disease-specific 16S microbiota identified in the samples. Only significant (p-value ≤0.05) are displayed. Color saturation shows that the numeric correlation p-value was < 0.05 in all cases.

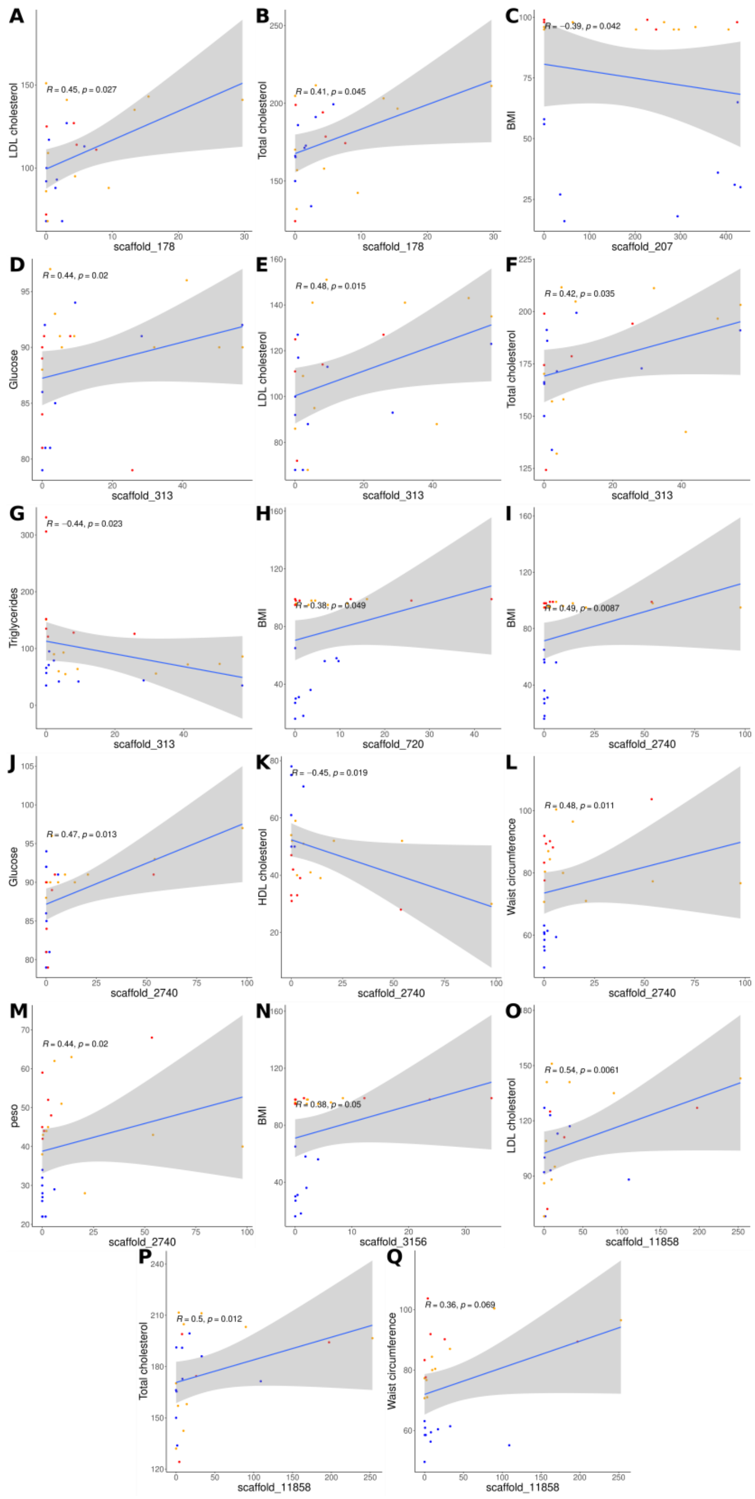

Figure S18. Linear regression and Spearman correlation of contigs that significantly correlated with disease-specific clinical and anthropometrical parameters, Related to Figure 6B. The title of each graph corresponds to each correlation. The y-axis shows the value of the clinical and anthropometrical parameters, and the x-axis shows the abundance in RPKM for each contig. Blue circles = NW samples; orange circles = O samples; and red circles = OMS samples.

Samples ID	Gender	Age		Samples ID	Gender	Age
NW_2	Male	7	**VS**	OMS_2	Male	7
NW_4	Male	7	**VS**	OMS_4	Male	7
NW_10	Female	7	**VS**	O_7	Female	7
NW_3	Female	8	**VS**	O_8	Female	8
NW_8	Male	8	**VS**	O_5	Male	8
NW_1	Female	9	**VS**	O_3	Female	9
NW_6	Female	9	**VS**	OMS_3	Female	9
NW_7	Male	9	**VS**	O_2	Male	9
NW_9	Male	9	**VS**	O_4	Male	9
NW_5	Male	10	**VS**	OMS_6	Male	10
O_6	Male	10	**VS**	OMS_8	Male	10
O_10	Male	9	**VS**	OMS_5	Male	9
O_9	Male	8	**VS**	OMS_1	Male	9
OMS_7	Female	10	**VS**	O_1	Male	10

Table S1. Mexican children cohort paired by age, gender and health status, Related to STAR methods. NW: Normal Weight, O: Obese, OMS: Obese with Metabolic Syndrome; VS: versus.

Samples ID	Gender	Age	Weight (Kg)	Height (cm)	BMI (percentile)	WC (cm, percentile)	BP (mmHg, percentile)	Glucose (mg/dL)	TG (mg/dL)	HDL-cholesterol (mg/dL)
**NW_118**	Female	9	34	142	56th	58.5, 25th	95/70, 23th/79th	81	79	50
**NW_119**	Male	7	26	128.6	58th	56.3, 25th	98/65, 40th/69th	92	35	61
**NW_120**	Female	8	29	134	56th	59.4, 25th	91/66, 19th/72th	91	44	71
**NW_010**	Male	7	22	121.3	31th	61.4, 25th	89/67, 26th/80th	81	95	50
**NW_124**	Male	10	32	141.2	30th	60.9, 25th	100/75, 38th/88th	86	57	54
**NW_147**	Female	9	30	132.3	65th	63.1, 25th	89/60, 14th/51th	79	66	61
**NW_161**	Male	9	28	134	36th	60.4, 25th	97/64, 38th/64th	94	42	78
**NW_169**	Male	8	27	134	27th	58.5, 25th	99/71, 45th/82th	92	71	50
**NW_193**	Male	9	22	122.2	18th	55.1, 25th	92/62, 38th/64th	85	42	75
**NW_314**	Female	7	22	124.5	15th	49.6, 25th	86/61, 14th/61th	81	35	75
**O_121**	Male	10	43	137.6	95th	80.4, >75th	100/68, 44th/74th	90	55	52
**O_122**	Male	9	44	138.5	96th	87, >90th	99/62, 37th/52th	90	56	59
**O_123**	Female	9	62	139.9	99th	100.4, >90th	100/72, 42th/85th	90	86	51
**O_152**	Male	9	63	153.5	98th	96.5, >90th	113/68, 71th/64th	90	73	39
**O_039**	Male	8	40	137	95th	76.7, >90th	86/54, 7th/27th	97	90	30
**O_418**	Male	10	51	148.6	96th	80, > 75 th	117/57, 84th/32th	91	64	41
**O_420**	Female	7	28	119.9	95th	71, >90th	82/61, 10th/63th	91	93	52
**O_434**	Female	8	38	135.7	95th	70.7, > 75 th	105/59, 64th/46th	88	151	54
**O_445**	Male	8	43	137.3	98th	77.3, >90th	120/79, 95th/94th	93	60	52
**O_090**	Male	9	45	143.2	95th	84.4, >90 th	91/64, 11th/57th	96	72	40
**OMS_124**	Male	9	59	146.1	98th	91.9, >90th	124/80, 96th/93th	81	135	47
**OMS_125**	Male	7	52	136.4	99th	90.2, >90th	98/62, 32th/54th	89	152	33
**OMS_126**	Female	9	44	131.2	98th	89.4, >90th	88/60, 14th/53th	79	126	42
**OMS_288**	Male	7	48	136.5	99th	88.2, >90th	104/72, 55th/83	91	128	39
**OMS_446**	Male	9	45	133.2	98th	83.3, >90th	119/78, 96th/94th	90	306	33
**OMS_055**	Male	10	55	142.5	98th	95.6, >90th	88/60, 7th/46th	91	276	24
**OMS_064**	Female	10	42	136	95th	77.6, >75th	99/68, 41th/76th	84	331	31
**OMS_087**	Male	10	68	149.2	99th	103.7, >90th	100/61, 28th/45th	91	121	28

Table S2. Baseline characteristics of the Mexican children cohort, **Related to STAR methods**. BMI: Body mass index; WC: Waist circumference; BP: Blood pressure; TG: Triglycerides; HDL: High density lipoprotein.

Samples ID	VLP's for each triplicate	VLP's count for five fields					VLP's counts average for sample	VLP´s for 10µl of sample	VLP´s per 250 mg of feces	VLP's per gram of feces
		1	2	3	4	5				
	1	1355	964	1003	1496	1147				
NW-10	2	1760	2506	920	1146	988	1211	15536178	310723563	1242894254
	3	927	1014	707	1086	1147				
NW-118	1	13959	17957	14118	10872	14623	14811	190003029	3800060579	15200242316
NW-119	2	14892	18204	17755	16629	18793				
NW-120	3	10511	17228	14964	10670	10990				
	1	4339	2210	3779	3081	3530				
NW-124	2	3666	3028	4233	1518	2933	3041	39006361	780127216	3120508864
	3	2210	2799	3442	2579	2262				
	1	540	802	185	891	8110				
NW-147	2	643	1494	640	586	678	1371	17591305	351826102	1407304410
	3	1079	1055	833	1606	1427				
	1	579	556	1835	1811	378				
NW-161	2	1218	345	403	315	111	1033	13252237	265044746	1060178984
	3	579	6479	1835	1811	378				
	1	1305	3777	2405	3092	2969				
NW-169	2	5708	8132	15074	5982	1813	4606	59083831	1181676615	4726706459
	3	5708	8132	1795	1774	1419				
	1	3485	2630	2048	1389	2978				
NW-193	2	2394	1571	1821	1606	1867	1947	24980526	499610512	1998442049
	3	2124	1367	892	1570	1467				
	1	1315	178	2850	4107	3366				
NW-314	2	2978	1230	1886	1612	2083	1932	24781256	495625122	1982500490
	3	253	598	839	1349	4332				
O-121	1	41499	47671	50919	48380	43696				
O-122	2	49528	51804	51156	51744	42080	46771	600002138	12000042762	48000171046
O-123	3	44412	45300	43321	42641	47411				
	1	5718	5498	5126	3636	7262				
O-152	2	2806	8352	4261	2232	3539	4159	53349488	1066989755	4267959020
	3	2309	3322	2914	1576	3829				
	1	1421	2999	2963	3369	4469				
O-39	2	4013	1710	1362	2581	2224	2362	30299225	605984499	2423937996
	3	2587	1209	1002	1959	1560				
	1	878	769	861	2482	1089				
O-418	2	525	1319	884	747	541	1130	14493648	289872962	1159491849
	3	1213	2275	1806	469	1089				
	1	3314	1740	2308	1864	1866				
O-420	2	5933	1238	1357	1426	1862	1995	25586886	511737728	2046950913
	3	1115	828	1671	1534	1862				
	1	2574	271	830	678	1565				
O-434	2	716	741	830	678	2563	952	12216160	244323207	977292829
	3	448	351	418	1106	515				
	1	296	750	1069	966	1688				
O-445	2	928	1086	895	1225	1174	1124	14420098	288401960	1153607840
	3	1869	1238	1023	966	1688				
	1	178	3620	9833	4006	790				
O-90	2	784	6203	2946	6779	2563	3195	40981096	819621915	3278487661
	3	811	752	1903	939	5811				
OMS-124	1	29725	32245	30969	37031	34731				
OMS-125	2	37130	31979	35136	32670	36854	33519	429998753	8599975056	34399900222
OMS-126	3	38089	29937	30916	35198	30176				
	1	3381	4479	2692	4097	5630				
OMS-288	2	2423	2086	3132	2321	2691	2752	35307474	706149488	2824597951
	3	1476	2086	2053	1416	1321				
	1	1080	556	721	1684	662				
OMS-446	2	731	363	527	705	558	870	11155670	223113408	892453630
	3	279	751	888	579	2960				
	1	396	660	1512	568	1101				
OMS-55	2	560	2155	920	465	818	833	10685292	213705835	854823341
	3	510	1077	610	588	554				
	1	1487	1265	1945	874	15131				
OMS-64	2	352	345	304	874	920	1999	25646753	512935056	2051740223
	3	1487	1265	1945	874	920				
	1	3315	3927	2659	3118	3449				
OMS-87	2	2570	2451	1701	2560	2219	2504	32119163	642383252	2569533007
	3	1120	1427	1963	1628	3449				

Table S3. VLPs counts, Related to Figure 1. The number of VLPs was determined in each field (total: five fields) by triplicate for every sample.

Assembly	18,602 contigs assembly	12,287 contigs assembly	4,611 phage contigs assembly
# contigs (>= 0 bp)	18602	12287	4611
# contigs (>= 1000 bp)	18602	12287	4611
# contigs (>= 5000 bp)	8760	8760	3445
# contigs (>= 10000 bp)	2621	2621	1158
# contigs (>= 25000 bp)	364	364	184
# contigs (>= 50000 bp)	69	69	40
Total length (>= 0 bp)	127030619	105263498	43100311
Total length (>= 1000 bp)	127030619	105263498	43100311
Total length (>= 5000 bp)	89542009	89542009	37896750
Total length (>= 10000 bp)	47836069	47836069	22217717
Total length (>= 25000 bp)	14977436	14977436	7975753
Total length (>= 50000 bp)	5068871	5068871	3137340
# contigs	18602	12287	4611
Largest contig	176210	176210	176210
Total length	127030619	105263498	43100311
GC (%)	49.69	49.7	49.71
N50	7480	9097	10370
N75	4615	5841	6332
L50	4450	3125	1093
L75	9954	6797	2452
# N's per 100 kbp	0.05	0.05	0

Table S5. Quast analysis of the viral assemblies, Related to STAR methods (de novo contig assembly).

Sample-Id	Disease-Type	Reads_remaining_postQuality-Filtered (paired_seq)	Quality-reads mapped to the 18,602 contigs assembly	% of mapped reads to the 18,602 contigs assembly	Quality-reads mapped to the 12,287 contigs assembly	% of mapped reads to the 12,287 contigs assembly	Quality-reads mapped to the 4,611 phage contigs assembly	% of mapped reads to the 4,611 phage contigs assembly
NW_118	Normal Weight	6483370	3446174	53.15	3000980	46.29	1723270	57.42
NW_119	Normal Weight	4105736	1964917	47.86	1680101	40.92	703738	41.89
NW_120	Normal Weight	3930198	2020079	51.40	1689329	42.98	663764	39.29
NW_10	Normal Weight	1415490	1043862	73.75	1021513	72.17	544750	53.33
NW_124	Normal Weight	3873168	3127816	80.76	3084258	79.63	1600132	51.88
NW_147	Normal Weight	3777848	2720684	72.02	2674804	70.80	1407664	52.63
NW_161	Normal Weight	4883020	3413536	69.91	3350099	68.61	1780477	53.15
NW_169	Normal Weight	299550	170192	56.82	159755	53.33	92395	57.84
NW_193	Normal Weight	4007412	2338006	58.34	2174355	54.26	1093916	50.31
NW_314	Normal Weight	945188	733140	77.57	728349	77.06	379352	52.08
OB_121	Obese	6222168	3160537	50.79	2777058	44.63	1379468	49.67
OB_122	Obese	3390676	1478086	43.59	1234603	36.41	575184	46.59
OB_123	Obese	714182	242360	33.94	203736	28.53	110118	54.05
OB_152	Obese	5213758	2212411	42.43	2032799	38.99	1536439	75.58
OB_39	Obese	2377282	1336861	56.23	1273595	53.57	691467	54.29
OB_418	Obese	933454	647537	69.37	631942	67.70	318605	50.42
OB_420	Obese	1345222	726624	54.02	686337	51.02	343508	50.05
OB_434	Obese	945650	679514	71.86	667387	70.57	350182	52.47
OB_445	Obese	1599244	982795	61.45	946904	59.21	495001	52.28
OB_90	Obese	699572	282616	40.40	263250	37.63	126100	47.90
OMS_124	Obese with Metabolic Syndrome	2319728	1376347	59.33	1240376	53.47	442514	35.68
OMS_125	Obese with Metabolic Syndrome	5190602	2889218	55.66	2471914	47.62	1004408	40.63
OMS_126	Obese with Metabolic Syndrome	4555974	2352597	51.64	2066334	45.35	828660	40.10
OMS_288	Obese with Metabolic Syndrome	1229670	481883	39.19	410001	33.34	175817	42.88
OMS_446	Obese with Metabolic Syndrome	817376	661852	80.97	656773	80.35	345680	52.63
OMS_55	Obese with Metabolic Syndrome	1641244	1077006	65.62	1032205	62.89	515729	49.96
OMS_64	Obese with Metabolic Syndrome	1497064	929203	62.07	866113	57.85	445543	51.44
OMS_87	Obese with Metabolic Syndrome	445510	281515	63.19	270091	60.63	138128	51.14

Table S6. Number of reads mapped to the contig assemblies, Related to Figure S7 and STAR methods.
